# Biowaste‐Derived Catalysts for Sustainable Electrochemical Water Splitting: A Pathway to Circular Bioeconomy

**DOI:** 10.1002/cssc.70718

**Published:** 2026-05-18

**Authors:** Vishal P. Bhandigare, Jaydip K. Sawant, Sourabh B. Ghode, Jihyeon Kim, Chandrashekhar S. Patil, Charalampos Pitsalidis, Kyungsoon Park, Jinho Bae

**Affiliations:** ^1^ Department of Chemistry and Cosmetics Jeju National University Jeju Republic of Korea; ^2^ Department of Ocean System Engineering Jeju National University Jeju Republic of Korea; ^3^ Department of Physics Khalifa University Abu Dhabi UAE

**Keywords:** biowaste‐derived carbon catalysts, circular bioeconomy, electrochemical water splitting, sustainable hydrogen generation, transition‐metal‐based catalysts

## Abstract

The accelerating global pursuit of carbon neutrality has intensified the need for sustainable, low‐cost hydrogen‐production technologies. Electrochemical water splitting, driven by renewable electricity, offers a clean pathway for hydrogen generation; however, large‐scale deployment is hindered by the high cost, scarcity, and limited durability of noble‐metal catalysts. In response, biowaste‐derived materials have emerged as a sustainable solution, transforming agricultural, food, and marine residues into high‐value electrode architectures. Naturally enriched with carbon frameworks and heteroatoms (N, S, P, B), such wastes can be converted through pyrolysis, activation, or templated synthesis into heteroatom‐doped porous carbons and biocarbon‐supported transition‐metal hybrids with abundant defects and accelerated charge transport. These tailored electrodes deliver competitive activity for both the hydrogen evolution reaction and oxygen evolution reaction, approaching Pt and IrO_2_ benchmarks while offering sustainability and scalability. This review consolidates conversion strategies, structural design principles, and catalytic mechanisms, emphasizing heteroatom modulation, metal–carbon interface engineering, and hierarchical morphology. It further establishes a structure‐property‐performance framework linking precursor chemistry, conversion route, and electrode architecture to catalytic behavior, while addressing fabrication strategies, benchmarking protocols, degradation mechanisms, and techno‐economic relevance. By integrating waste valorization with green electrocatalysis, biowaste‐derived electrodes offer a promising pathway toward circular, low‐carbon hydrogen‐energy systems.

## Graphical abstract

Overview of biowaste‐derived electrocatalysts, including precursor selection, conversion and fabrication strategies, structure–activity relationships, and hydrogen evolution reaction/oxygen evolution reaction/overall water‐splitting performance for sustainable hydrogen production.

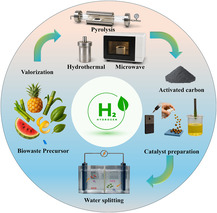

## 1 Introduction

Rising global energy demand and the need to decarbonize major sectors have intensified the pursuit of sustainable, carbon‐neutral fuels [1, 2]. In this context, owing to its exceptional energy density, inherently clean combustion profile, and renewable production pathways, hydrogen has rapidly emerged as a pivotal and transformative energy vector within the evolving low‐carbon landscape [1, 3, 4, 5]. As a result, serving as a central link between renewable power and energy‐intensive industries, the hydrogen economy supports storage, conversion, and transport across the entire energy value chain [6]. Global forecasts predict soaring low‐carbon hydrogen demand, making it vital for net‐zero goals [7, 8, 9, 10, 11, 12, 13]. Therefore, large‐scale renewable hydrogen generation is a core priority for the global energy transition [14, 15]. Within this broader framework, electrochemical water splitting (EWS) offers a pristine and future‐defining pathway for hydrogen production by converting electricity into chemical energy without generating carbon dioxide or combustion‐derived pollutants, establishing it as a cornerstone technology for sustainable fuel synthesis [16, 17, 18, 19]. Fundamentally, this process involves the hydrogen evolution reaction (HER) at the cathode and the oxygen evolution reaction (OER) at the anode, both of which are hindered by sluggish multi‐electron transfer and complex bond‐breaking/bond‐forming kinetics, necessitating highly efficient catalysts to surmount these intrinsic barriers [20, 21]. In addition to its clean chemistry, its modular architecture and compatibility with renewable power enable operation under mild and environmentally benign conditions [22, 23]. Although the theoretical minimum voltage for water splitting is 1.23 V, additional *η* is required in practical systems due to kinetic and thermodynamic limitations [24, 25]. For this reason, current research focuses on minimizing this *η* and enhancing overall efficiency through advanced catalyst engineering and rational electrode design [6, 14, 26, 27, 28, 29].

Despite major progress, the most efficient HER and OER catalysts remain noble metals like Pt, IrO_2_, and RuO_2_ [30]. Although these catalysts provide high activity and acceptable initial stability, they also face important practical drawbacks such as, they are expensive, limited in natural supply, and tend to lose performance under acidic or alkaline operating conditions due to dissolution, surface changes, and gradual chemical instability [31, 32, 33, 34, 35, 36]. Their restricted availability and price fluctuations further limit their suitability for large‐scale water electrolysis [30]. Hence, research now focuses on earth‐abundant alternatives such as transition‐metal compounds and heteroatom‐doped carbons, which not only offer comparable activity at lower cost but also provide benefits such as adjustable electronic structure, larger numbers of active sites, improved chemical durability, and compatibility with scalable synthesis methods [37, 38, 39, 40, 41]. Recent advances in tuning electronic structure, defects, and interfaces have further improved their catalytic efficiency [42, 43]. However, despite their promise, many transition‐metal and heteroatom‐doped carbon catalysts still require costly precursors and complex synthesis steps, highlighting the need for more accessible, low‐cost alternatives such as biowaste‐derived materials [31]. As a promising strategy for sustainable hydrogen‐production technologies, low‐cost catalysts are the valorization of biowaste and biomass residues [44, 45]. Agricultural, marine, and food wastes rich in C, N, S, and P can yield heteroatom‐doped carbons with tunable conductivity, porosity, and active sites [46, 47, 48, 49]. Through carbonization, hydrothermal processing, or plasma activation, these materials undergo structural transformation from natural organic matter into porous carbon frameworks containing micro‐ and mesopores [50, 51, 52, 53, 54, 55]. During these activation steps, volatile components decompose, heteroatoms are partially released, and the carbon skeleton reorganizes, creating interconnected voids and defect‐rich channels within the matrix [56, 57, 58]. These interconnected pores increase the accessible surface area, shorten ion‐transport pathways, and support faster mass movement during HER and OER [59, 60, 61, 62]. When combined with transition‐metal salts or oxides through in situ growth, they form conductive hybrid catalysts with well‐distributed and stable metal sites [63, 64, 65, 66, 67]. This approach converts low‐value waste into useful electrocatalysts, supporting both environmental protection and circular bioeconomy [6, 49, 68, 69, 70, 71].

Recent studies show that biowaste‐derived carbons possess naturally occurring defects, abundant functional groups, and tunable surface chemistry that collectively enhance HER activity by facilitating charge transfer and creating favorable hydrogen adsorption sites [45, 59, 72, 73, 74, 75]. Likewise, when biomass‐derived carbons are combined with transition‐metal oxides, phosphides, or sulfides, they form hybrid electrocatalysts that deliver efficient OER with lower *η* and improved durability [71, 76, 77, 78]. The hierarchical pores and high graphitic conductivity characteristics of these materials further promote rapid gas diffusion and effective charge transport, supporting stable long‐term electrolysis [45, 79, 80]. Moreover, these systems establish a closed‐loop route in which waste resources are upcycled into functional, value‐added catalysts, thereby reducing dependence on critical metals and lowering environmental impact [6, 79]. Together, these findings position biowaste‐derived electrocatalysts as a practical and sustainable platform for high‐performance water splitting, offering a balanced combination of activity, durability, and affordability while aligning with the broader goals of circular and environmentally responsible hydrogen production [6, 59].

While the fabrication of biomass‐derived catalysts has become a highly attractive topic, existing reviews frequently serve as mere summaries of isolated electrochemical processes, lacking a cohesive theoretical and practical framework [6, 59, 81, 82, 83, 84, 85]. To transcend simple literature aggregation, a comprehensive structure‐to‐property framework is established in this review, fundamentally linking the intrinsic characteristics of diverse biowaste precursors directly to the properties of the final electrocatalysts [85]. Unlike previous works, the analysis extends beyond apparent catalytic activity to provide a dedicated, reaction‐specific comparison of the distinct demands for HER versus OER [86]. Furthermore, in‐depth mechanistic insights into long‐term degradation behaviors are presented, with a specific focus on critical issues such as heteroatom leaching and carbon matrix corrosion under high oxidative potential. Crucially, the “circular bioeconomy” concept is substantiated through the integration of a quantitative techno‐economic cost analysis, wherein the scalability, energy consumption, and pricing of biowaste‐derived materials are directly compared against traditional noble and transition‐metal benchmarks. By bridging theoretical design principles, rigorous degradation mechanics, and economic realities, biowaste‐derived electrocatalysts are positioned herein not merely as green alternatives, but as technically and economically viable replacements for conventional water electrolysis systems [87, 88].

## 2 Fundamentals of EWS

**FIGURE 1:**
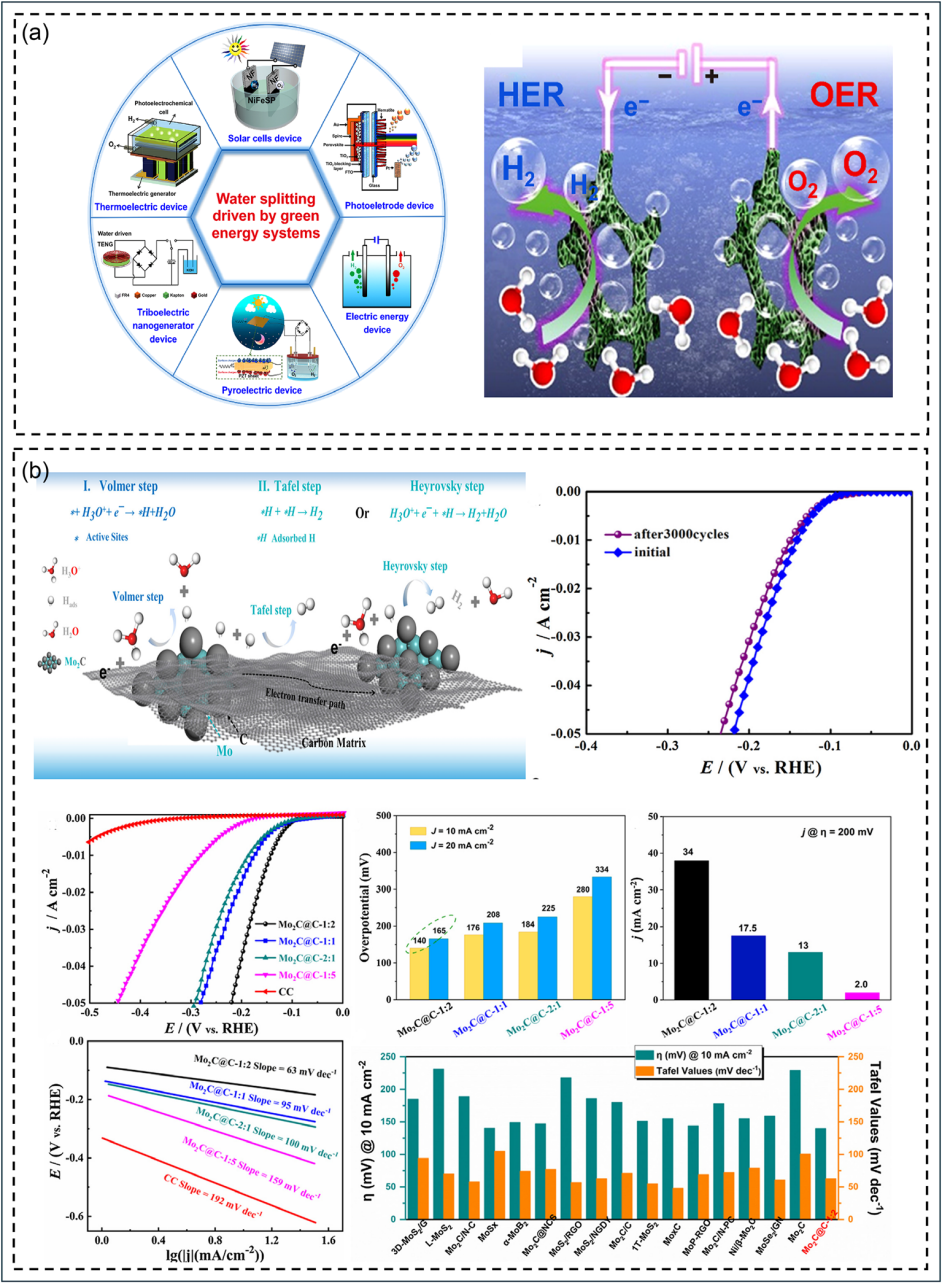
Fundamental principles and evaluation framework of EWS (a) Water splitting driven by green energy systems and schematic representation of overall water splitting (OWS) in a two‐electrode configuration showing HER and OER processes. Reproduced with permission [89]. Copyright 2020, Springer Nature. (b) Representative overall water‐splitting evaluation illustrating reaction mechanism, polarization curves, Tafel analysis, *η*, and cycling stability for reliable benchmarking of electrocatalytic performance. Reproduced with permission [90]. Copyright 2020, Elsevier.

EWS couples the HER and OER to achieve 2H_2_O → O_2_ + 2H_2_ (E^°^ = 1.23 V vs. SHE), as shown in Figure 1a [89, 91]. Reliable evaluation requires controlled catalyst preparation, cell design, kinetic analysis, and standardized benchmarking across pH and cell types [3, 36, 91, 92]. Community protocols assess activity, stability, and Faradaic efficiency (FE), recommending overpotential (*η*) reporting at 10 mA cm^−2^, gas verification, and short‐term durability tests [93, 94]. Best practices specify consistent reporting of Tafel slope, electrochemically active surface area (ECSA), iR correction, electrolyte, and conditions [18, 93]. All potentials should be referenced to the reversible hydrogen electrode (RHE) for reproducibility [2]. In this framework, *η* is derived from LSV/CV polarization curves at defined current densities with proper iR‐drop correction to avoid artifacts [93]. Tafel plots of *η* versus log j identify linear kinetic regions to extract slopes and rate‐determining steps [95]. Activities are normalized by ECSA or mass to ensure fair comparison, with normalization methods clearly stated. Turnover frequency (TOF) should be reported with explicit site assumptions and uncertainties [93]. FE verification via H_2_/O_2_ gas analysis confirms true catalytic activity and stability, with detailed protocols now available for accurate FE quantification and error analysis [96, 97].

Electrochemical impedance spectroscopy (EIS) is used to distinguish charge–transfer, ion‐transport, and mass‐transport effects under realistic conditions [98]. Geometry and hydrodynamics such as film thickness, ink composition, and bubble dynamics strongly influence apparent activity and durability in OER benchmarking and RDE/GDE studies [99, 100]. As shown in Figure 1b, representative LSV, Tafel, and stability plots demonstrate how proper correction and rigorous methodology yield reliable benchmarking for overall water‐splitting evaluation [90]. Over time, community standards have evolved from basic benchmarking defining current‐density targets, FE verification, and short stability tests to detailed reporting protocols with clear reference scales [2, 85, 93, 99, 100]. These advances now include iR correction, ECSA/TOF normalization, and artifact control, culminating in standardized OER benchmarking under model conditions with mapped substrate and stress‐test effects and clarified RDE pitfalls like bubble blockage and passivation [101]. Today, authors are expected to report cell type, reference calibration, electrolyte composition, temperature, rotation or flow conditions, ink and substrate preparation, and gas handling, as these parameters critically affect measured kinetics and durability [91].

These principles enable consistent evaluation of biowaste‐derived electrocatalysts for HER and OER. Recent reviews stress that circular‐economy materials such as heteroatom‐doped carbons, carbon‐metal hybrids, and multicomponent systems must follow the same benchmarking standards: report *η* at defined current densities with iR correction, derive Tafel slopes from linear regions, present ECSA‐ or TOF‐normalized activities with stated assumptions, verify FE via gas analysis, and confirm stability through transparent stress testing to enable valid comparison with commercial Pt/C and Ir/Ru oxide catalysts [102, 103, 104]. Rigorous benchmarking is essential for credibly demonstrating that biowaste‐derived catalysts can match or even surpass the performance of state‐of‐the‐art Pt/C and Ir/Ru‐oxide benchmarks on an intrinsic basis rather than through experimental artifacts [105].

## 3 Biowaste‐Derived Precursors: Classification, Conversion, and Structure–Property Relationships

Biowaste streams are abundant, low‐cost, and compositionally rich sources for functional carbon materials used in energy, environmental, sensing, and medical applications [59, 106]. Their main components, including cellulose, lignin, proteins, and inorganic minerals, provide natural heteroatom sources and structural templates for producing porous and doped carbon frameworks [107, 108]. Biowaste resources can be broadly classified into agricultural, food, marine, lignocellulosic, and specialized waste categories [106, 108, 109]. Agricultural residues such as rice husk, straw, and bagasse are widely employed to produce porous carbons, where the silica‐rich composition of rice husk is particularly advantageous for generating hierarchical pore structure [110]. Food wastes, including fruit peels, nutshells, and coffee grounds, are attractive precursors for high‐surface‐area and nitrogen‐doped carbons [111, 112, 113]. Marine‐derived wastes such as shells and seaweeds provide nitrogen‐, sulfur‐, and mineral‐rich frameworks, which are beneficial for catalytic applications [114, 115]. Lignocellulosic biomass and specialized wastes (e.g, animal bones and sewage sludge) contribute aromatic carbon frameworks and mineral‐derived catalytic sites [116]. The selection of biomass feedstock is generally governed by heteroatom content, ash composition, local availability, cost, and environmental impact [117]. Compared with carbons derived from synthetic polymers, resins, or metal‐organic framework (MOF) templates, biowaste precursors offer a unique combination of intrinsic heteroatom content, pre‐existing hierarchical structure, and ultralow feedstock cost, which substantially reduces the overall material and processing footprint of high‐performance electrocatalysts.

Owing to these advantages, biomass‐derived electrocatalysts are increasingly explored as sustainable, cost‐effective, and high‐performance alternatives to noble metal catalysts (e.g, Pt/C) for energy conversion and storage reactions such as oxygen reduction reaction (ORR), OER, and HER [6, 14, 59, 118, 119]. Biomass serves as an abundant and renewable carbon source that inherently contains heteroatoms such as N, P, S, and O [80, 119, 120]. During thermal conversion (e.g, pyrolysis or hydrothermal carbonization (HTC)), these heteroatoms can be incorporated into the carbon lattice, yielding heteroatom‐doped porous carbons with tunable electronic structure and catalytic activity [80, 120, 121]. The effectiveness of biomass‐derived electrocatalysts is strongly influenced by the physicochemical properties of the raw precursor. Many plant‐based biowastes exhibit naturally interconnected hierarchical porosity (macro‐, meso‐, and micropores), which can be preserved after carbonization and provides efficient pathways for electrolyte penetration and ion diffusion [6, 80, 122, 123]. In addition, biological tissues contain proteins, amino acids, and chlorophyll derivatives that enable intrinsic self‐doping, particularly with nitrogen, sulfur, and phosphorus, which improves conductivity and modifies surface adsorption properties [6, 59, 124]. The lignocellulosic composition of biomass also plays a key role, as lignin‐rich feedstocks often yield carbon frameworks with higher aromaticity and improved conductivity, while cellulose‐rich materials favor the formation of three‐dimensional porous networks [125, 126]. Furthermore, biomass‐derived carbons typically retain oxygen‐containing functional groups (e.g, hydroxyl, carbonyl, and carboxyl groups), which enhance hydrophilicity and provide anchoring sites for metal species [80, 125]. Overall, these precursor‐dependent features translate directly into structure–property relationships that govern electrocatalytic performance. Specifically, heteroatom doping modulates the electronic structure and optimizes adsorption energetics for HER, while hierarchical porosity and morphology facilitate mass transport and electrolyte accessibility, which are critical for OER [127, 128, 129]. In addition, surface functional groups act as active centers and anchoring sites that regulate metal nucleation, dispersion, and metal–support interactions, thereby enhancing catalytic activity and long‐term stability in transition‐metal‐based systems. These correlations between biomass chemistry, microstructure, and catalytic behavior have been widely demonstrated, highlighting that biomass should be regarded not only as a sustainable precursor but also as a key design parameter for rationally engineering efficient and scalable electrocatalysts for hydrogen production [6, 47, 119, 121].

**FIGURE 2:**
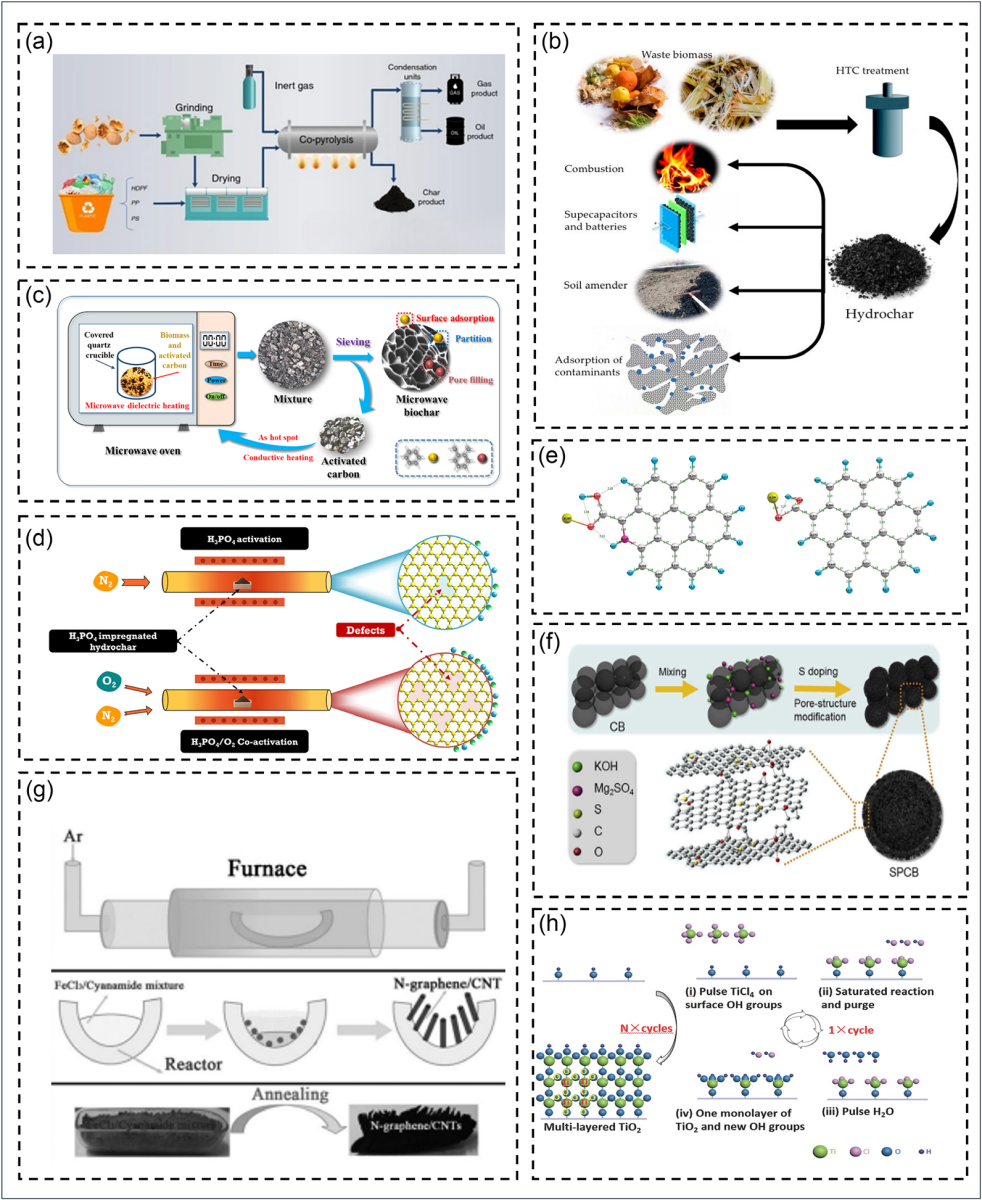
Schematic illustration of various synthesis and modification strategies for biowaste‐derived carbon materials (a) co‐pyrolysis process for activated carbon production. Reproduced with permission [131]. Copyright 2023, Springer Nature. (b) HTC and hydrochar applications. Reproduced with permission [132]. Copyright 2020, MDPI. (c) microwave‐assisted pyrolysis for rapid biochar formation. Reproduced with permission [133]. Copyright 2022, Elsevier. (d) activation and co‐activation strategies for tuning porosity and surface defects. Reproduced with permission [134]. Copyright 2025, Elsevier. (e) optimized structures showing heteroatom‐enhanced sulfur adsorption. Reproduced with permission [135]. Copyright 2013, Wiley. (f) heteroatom doping and pore modulation in carbon frameworks. Reproduced with permission [136]. Copyright 2021, Wiley. (g) synthesis mechanism of N‐graphene/CNT hybrids via annealing. Reproduced with permission [137]. Copyright 2014, Wiley. (h) ALD process illustrating sequential oxide layer formation on reactive substrates. Reproduced with permission [138]. Copyright 2016, Wiley.

To realize these properties in practice, biowaste is transformed into carbon materials through thermal and chemical methods that tune porosity, defect density, and graphitic order. Pyrolysis, the most common route, thermally decomposes biomass under N_2_ or Ar at 200°C–1000°C to produce amorphous or graphitic carbons [130]. As shown in Figure 2a, a fixed‐bed pyrolysis system enables controlled graphitic carbon formation under varied conditions [131]. Lower temperatures (200°C–600°C) retain oxygenated surface groups for adsorption, while higher (>800°C) enhance graphitization and conductivity [74]. Parameters like heating rate, dwell time, and temperature govern pore structure. HTC at 180°C–260°C efficiently converts wet biomass (e.g, food sludge, algae) into oxygen‐rich hydrochar precursors, as illustrated in Figure 2b [110, 132]. Microwave‐assisted biochar synthesis, shown in Figure 2c, uses dielectric heating of biomass with activated carbon, creating localized hot spots that accelerate carbonization and pore formation, yielding high‐surface‐area, porous biochars for environmental applications [133, 139].

Chemical activation using agents such as KOH, NaOH, H_3_PO_4_, or ZnCl_2_ at temperatures ranging between 600°C and 900°C introduces hierarchical porosity and significantly increases the specific surface area. For example, KOH activation promotes microporosity, while H_3_PO_4_ generates phosphate functionalities and mesoporosity (see Figure 2d) [134]. Physical activation and templating (CO_2_, steam, or silica templates) further enable tailored pore structures and ordered architectures. Hybrid or one‐pot approaches, combining HTC with simultaneous activation or doping, offer scalable synthesis routes by integrating multiple steps into a single process. The resulting microstructure directly affects device performance micropores (≤2 nm) enhance charge storage, mesopores (2–50 nm) facilitate ion transport, and graphitic domains ensure low impedance and rapid charge conduction. Therefore, achieving a balance between porosity, conductivity, and defect concentration is critical for optimizing materials for electrochemical and sensing applications [140].

The intrinsic heteroatoms in biomass enable natural doping for electronic and chemical tuning. Nitrogen (from proteins, chitin), sulfur (from amino acids), phosphorus (from bones, seeds), and oxygen (from polysaccharides, lignin) form pyridinic‐, pyrrolic‐, and graphitic‐N; thiophenic‐S; and phosphate or carbonyl O/P sites during carbonization [141]. N‐doping enhances conductivity and redox kinetics, while S‐doping modifies electronic structure; Jiangxuan Song et al. showed via density functional theory (DFT) that N promotes O–S bonding. Figure 2e shows the optimized structures of the sulfur adsorbed on –CO, or OH– groups in different types of N‐doped carbon [135]. P‐doping introduces hydrophilic, electron‐rich sites, improving charge transfer, demonstrated in Figure 2f through S‐doped carbon black for high‐capacitance electrodes [136]. Boron induces p‐type conductivity and defect tuning [111]. Moderate (400°C–700°C) and high (>800°C) carbonization favor functional and graphitic dopant forms, respectively [142], while agents like H_3_PO_4_ or thiourea enhance P/S doping and graphitization. Such doped carbons exhibit tuned electronic structure, high electrochemical activity, and strong adsorption for catalytic, sensing, and energy‐storage uses [143, 144]. Metal or metal oxide incorporation enables further tuning of physicochemical and electronic properties. In situ impregnation of metal salts (FeCl_3_, Co(NO_3_)_2_, Ni(NO_3_)_2_, Cu(NO_3_)_2_) into biomass before pyrolysis forms metallic, oxide, or M‐N‐C sites upon carbonization [145]. This one‐step process ensures strong metal–carbon coupling for enhanced redox and electrocatalytic activity. Figure 2g shows the setup and mechanism for N‐graphene/CNT hybrid synthesis with pre‐ and post‐annealing samples [137, 146]. Metal type and pyrolysis conditions determine the phase: Fe yields Fe‐N‐C for high ORR activity, while Co and Ni form spinel oxides (Co_3_O_4_, NiO) with superior capacitance. Fe, N‐doped NiCo_2_O_4_ catalysts use interface electron redistribution, where Fe and N doping improve water coordination and oxygen reactions; the Ni‐V‐O‐Fe structure boosts OER via multisite electron regulation [147].

Post‐synthesis modification deposits metal salts onto preformed carbons, followed by reduction or sol–gel treatment to form nanoparticles or oxide layers. Techniques like atomic layer deposition (ALD) and electrochemical deposition provide nanoscale control while maintaining porosity. Heteroatoms (N, O, P, S) anchor and stabilize metal species, preventing aggregation. Figure 2h shows an ALD process depositing multi‐TiO_2_ layers using TiCl_4_ and H_2_O on ‐OH‐functional substrates [138]. Metal‐carbon integration enhances charge transfer and interfacial synergy, boosting faradaic reactions and sensitivity, though excessive loading can block pores; thus, optimizing metal content and M‐N_*x*_ coordination is key for long‐term stability [148]. Comprehensive characterization ensures structure‐property validation. CHNS and XPS determine heteroatom states; BET, Raman, and XRD reveal porosity, defects, and phases. TEM/STEM shows morphology and coordination, while ICP measures metal content. Electrochemical (CV, EIS, GCD) tests evaluate charge transfer and redox activity [115]. N‐rich sources (chitin, coffee grounds, algae) enable N‐doping and M‐N‐C sites, silica‐rich rice husk yields mesoporosity. Moderate carbonization (500°C–700°C) preserves active groups; higher (800°C–1000°C) improves graphitization. Optimized activation, doping, and metal loading balance conductivity and reactivity, while scalable synthesis and stability testing ensure durable, eco‐friendly performance [132, 149].

In summary, Biowaste‐derived precursors represent a sustainable and adaptable method to produce functional carbon materials with specific structures and properties [49]. By selecting appropriate biomass sources and utilizing controlled processing techniques‐such as carbonization, chemical and physical activation, heteroatom doping, and hybridization‐researchers can create porous carbon frameworks that have optimized characteristics including surface area, pore distribution, defect density, and electronic conductivity [80, 119, 120]. These attributes are crucial for enhancing performance in various applications, such as electrochemical energy conversion (including water splitting and oxygen reduction reactions), energy storage (for supercapacitors and batteries), and sensing technologies. Recent studies indicate that carefully engineered biowaste‐derived carbons can achieve surface areas and levels of heteroatom doping that are on par with, or even surpass, those of commercial activated carbon and synthetic carbon materials derived from polymers or MOFs [120, 150, 151]. Moreover, these biowaste‐derived materials offer greater sustainability benefits as they utilize renewable and abundant feedstock sources.

To better illustrate the economic viability of this approach, a comparative cost analysis of precursor materials and associated synthesis expenses was conducted (Table 1). This analysis examined costs on a per kg catalyst basis, considering the amount of precursor needed, commercial chemical prices (sourced from Sigma–Aldrich's base catalog), and estimated energy consumption during thermal processing. The findings reveal that the primary determinant of the overall cost of biowaste‐derived carbon catalysts is the expense of activation and doping reagents, while the biowaste feedstock is considerably affordable and readily available [6, 80]. In contrast, noble‐metal catalysts incur significantly higher synthesis costs due to the exorbitant prices of Pt‐group metal precursors, even at minimal loadings [152, 153]. Transition‐metal‐based catalysts have moderate cost levels but still rely on refined metal salts and complex multistep synthesis processes, leading to increased chemical and processing costs [59, 152]. Overall, the cost comparison presented in Table 1 clearly indicates that biowaste‐derived catalysts offer a more economically viable solution for large‐scale electrochemical technologies, especially when considering their sustainability advantages and the scalability of their production.

**TABLE 1:** Comparative cost analysis for biowaste‐derived catalysts compared with conventional transition‐metal and noble‐metal catalysts. (Costs were estimated based on Sigma–Aldrich base catalog prices and the Korea‐average electricity rate).

Parameter	Biowaste‐Based Catalyst	Traditional Noble‐Metal Catalyst	Transition‐Metal Catalyst
Typical Precursor(s)	Agricultural/food biowaste (e.g, fruit peel, rice husk, spent coffee grounds)	Pt, Pd, Rh salts (e.g, H_2_PtCl_6_, PdCl_2_)	Fe, Co, Ni, Nitrates/Oxides
Market Price of Precursor (approx. USD/kg)	0−0.5	30,000–60,000	5–20
Cost of the amount used (USD/kg)	0−0.5	600–1200	1–4
Energy & Processing (USD/kg)	1–3	5–15	3–8
Activation/chemical agents (USD/kg)	0.3–1.0	0.2–0.6	0.2–0.6
Solvents/washing/filtration (USD/kg)	0.2–0.8	1.0–2.0	0.5–1.2
Total synthesis cost (USD/kg)	2–7	610–1240	6–20

## 4 Electrode Fabrication and Integration Strategies

### 4.1 Fabrication and Design Strategies for Biowaste‐Derived Binder‐Free Electrodes

**FIGURE 3:**
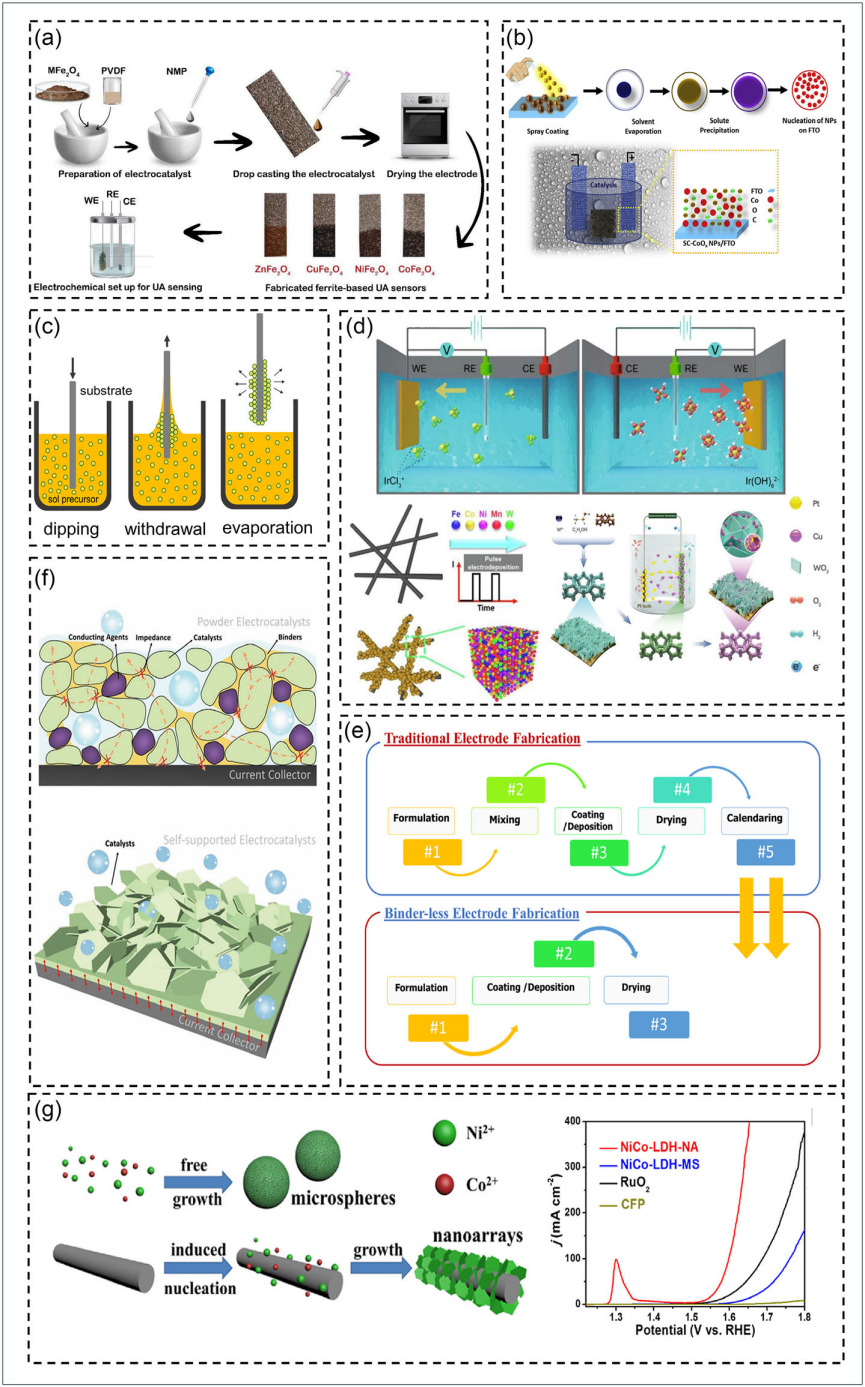
Schematic overview of common electrode fabrication techniques. Schematic illustration of the (a) drop‐casting. Reproduced with permission [154]. Copyright 2024, Elsevier. (b) spray‐coating. Reproduced with permission [155]. Copyright 2020, Elsevier. (c) dip‐coating. Reproduced with permission [156]. Copyright 2022, Springer Nature. (d) electrodeposition processes for electrode fabrication techniques. Reproduced with permission [157]. Copyright 2023, MDPI. (e) Schematic for the traditional and binder‐free electrode fabrication processes. Reproduced with permission [158]. Copyright 2024, Elsevier. (f) Schematic for the comparison of powder‐type and self‐supported electrocatalysts, and binder‐free, self‐supported architectures. Reproduced with permission [159]. Copyright 2021, Wiley. (g) Vertically aligned NiCo‐LDH nanoarray growth on carbon fiber paper. Reproduced with permission [160]. Copyright 2016, Elsevier.

Biowaste‐derived electrodes obtained from agricultural residues, fruit peels, or other biomass via pyrolysis, hydrothermal, or carbonization routes yield heteroatom‐doped carbons and carbon‐metal hybrids that act as sustainable catalysts for HER and OER. Their low cost, high activity, and eco‐friendly character make them attractive for water splitting, where fabrication strategies ensure strong electrical contact, mechanical robustness, and efficient mass transport. As shown in Figure 3a, drop casting involves depositing a homogeneous catalyst slurry onto conductive substrates such as glassy carbon or Ni foam (NF). After drying, it forms a uniform catalytic layer providing strong interfacial contact and stability; optimization of ink composition and solvent ratio markedly affects performance, and biochar coatings on NF enhance HER/OER efficiency by increasing porosity and active‐site exposure [154, 161, 162, 163, 164, 165, 166]. In Figure 3b, spray coating enables scalable, uniform films through atomized deposition of precursor solutions onto heated substrates, achieving precise thickness control and good adhesion [155, 167, 168, 169]. Spraying natural‐precursor inks onto NF yields binder‐free, porous electrodes with improved conductivity, gas diffusion, and mechanical strength [167, 170, 171]. Dip coating (Figure 3c) immerses substrates into precursor solutions followed by withdrawal and drying to form adherent films [156]. Ni‐Fe catalysts fabricated via dip‐coating and annealing exhibit low *η* and excellent durability on porous biowaste carbons [172, 173, 174, 175, 176]. Finally, electrodeposition (Figure 3d) electrochemically reduces metal ions from salt solutions onto conductive supports [157, 177, 178], allowing fine control of film thickness and morphology. It produces binder‐free, self‐supported electrodes with strong adhesion and superior conductivity; NiFe‐based electrodeposited films show large ECSAs and outstanding stability [179, 180, 181, 182].

Building on these fabrication foundations, binder‐free and self‐supported architectures eliminate insulating polymer binders and directly integrate catalytic layers onto conductive substrates, enhancing durability, conductivity, and active‐site accessibility [158, 183, 184]. The fabrication sequence for such electrodes is depicted in Figure 3e. As shown in Figure 3f, Yang et al. [159, 185, 186]. demonstrated that self‐supported electrocatalysts ensure robust electron pathways and structural integrity for large‐scale hydrogen production. Wang et al. further developed NiMo‐nitride nanowire arrays functioning as binder‐free bifunctional electrodes, achieving 10 mA cm^−2^ at 1.507 V with long‐term durability [186, 187, 188]. Likewise, Yu et al. designed vertically assembled NiCo‐layered double hydroxide (LDH) arrays on carbon–fiber paper (Figure 3g) where direct growth minimized binder‐induced resistance and enhanced OER activity [158, 160, 188, 189].

Collectively, these fabrication strategies including scalable deposition techniques and binder free or self‐supported architectures enhance electrocatalytic performance by improving electron transport pathways, increasing ECSA, and facilitating efficient mass transport of reactants and gas products [190, 191]. Binder free configurations generally exhibit lower interfacial resistance compared to slurry cast electrodes due to the absence of insulating polymer binders, which leads to more efficient charge transfer kinetics [158, 192, 193]. Among the discussed methods, electrodeposition and in situ growth approaches typically provide stronger interfacial adhesion and better structural stability, resulting in improved long‐term durability under electrochemical conditions. In contrast, drop casting and dip coating methods are often associated with weaker film adhesion and reduced stability, particularly during prolonged operation. Spray coating offers advantages in terms of uniform film formation and scalability, although its performance is strongly influenced by precursor dispersion and deposition parameters. These observations indicate that catalytic performance is closely related to the ability of each fabrication strategy to control conductivity, porosity, and mechanical integrity. However, variability in biowaste composition and limitations in achieving uniform large‐scale fabrication remain significant challenges affecting reproducibility. Therefore, future work should focus on developing standardized and scalable fabrication approaches that combine in situ growth with conductive self‐supported frameworks, enabling improved catalytic efficiency, durability, and practical applicability in sustainable hydrogen production systems [59, 81, 194].

### 4.2 Substrate‐Dependent Morphological and Structural Optimization for Enhanced Water‐Splitting Performance

**FIGURE 4:**
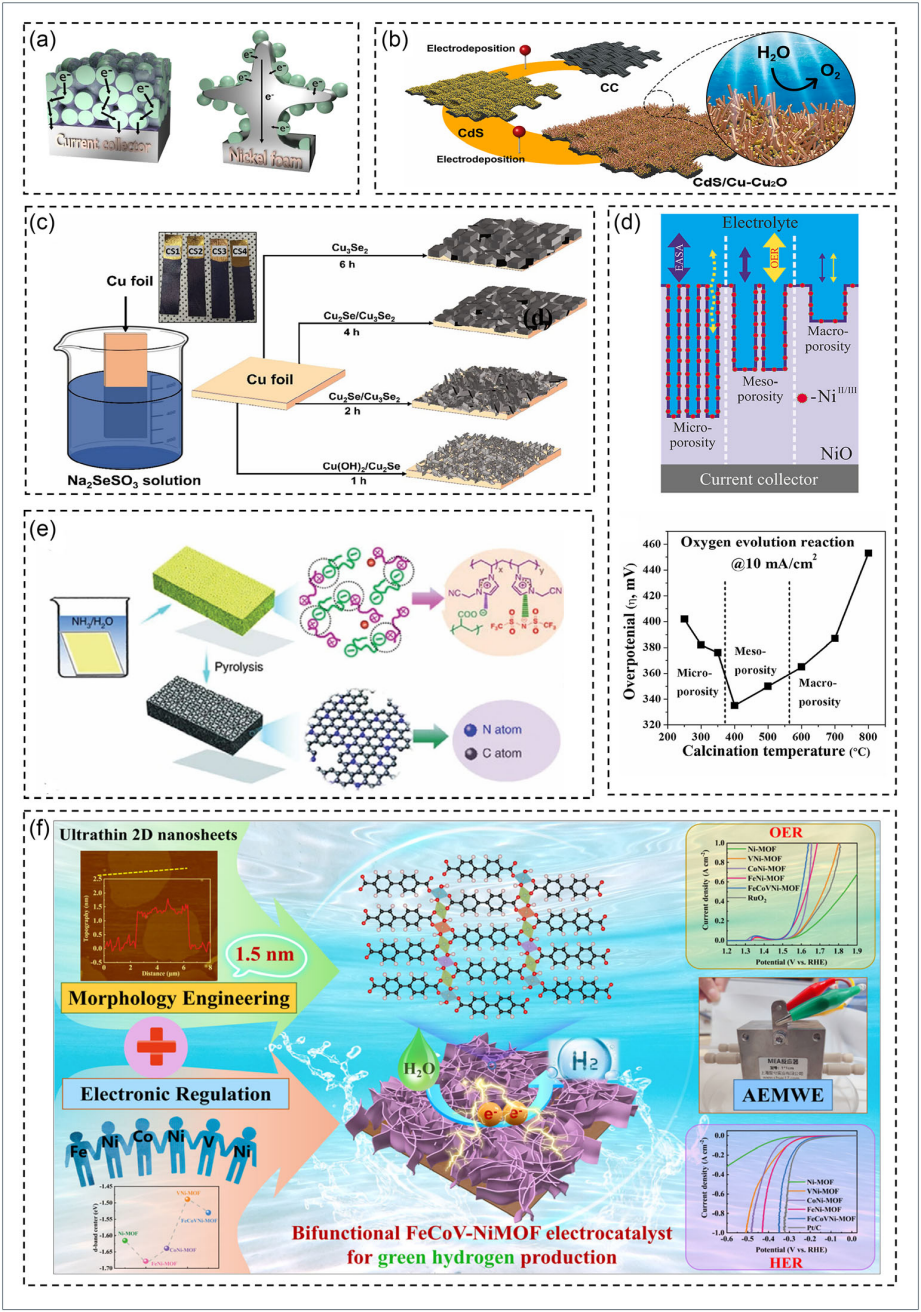
Overview of conductive substrates and morphology/structure‐engineered strategies for enhanced EWS: (a) comparison of powder catalysts and 3D Ni–foam electrodes for improved electron transport. Reproduced with permission [199]. Copyright 2017, RSC publication. (b) stepwise electrodeposition of CdS and Cu–Cu_2_O heterojunction photoanode fabricated on CC. Reproduced with permission [200]. Copyright 2025, Elsevier. (c) time‐dependent evolution of Cu‐Se films on copper foil through chemical bath deposition, highlighting the self‐grown Cu_3_Se_2_ conductive layer that strengthens interfacial adhesion, conductivity, and durability for efficient water splitting. Reproduced with permission [201]. Copyright 2023, Wiley. (d) micro to mesoporous NiO transition enabling better electrolyte diffusion and lower *η* of OER. Reproduced with permission [202]. Copyright 2022, Elsevier. (e) nitrogen‐doped porous carbon formed by NH_3_/H_2_O activation and pyrolysis. Reproduced with permission [203]. Copyright 2024, Wiley. (f) ultrathin FeCoV‐NiMOF nanosheet arrays with hierarchical porosity for boosted charge transfer and overall efficiency. Reproduced with permission [204]. Copyright 2025, Elsevier.

In electrochemical water‐splitting systems, the substrate or current collector serves as the foundational scaffold onto which electrocatalytic materials are directly grown or deposited; its primary functions include providing efficient charge transport, mechanical support, and a high accessible surface area for the active catalyst layer [159, 185, 195, 196, 197, 198]. Beyond simple mechanical support, biomass‐derived carbon materials and other conductive frameworks such as NF, Cu foil, and carbon cloth (CC) play a pivotal role in modulating charge transfer and active‐site accessibility. For example, NF has been extensively used as a three‐dimensional scaffold owing to its continuous porous framework and high conductivity, facilitating intimate catalyst‐substrate contact and improved electrocatalytic kinetics, as shown in Figure 4a [121, 199, 205]. Likewise, CC, with its flexible fibrous morphology, provides a large surface area for catalyst growth and enhanced electrochemical activity. As shown in Figure 4b, electrodeposition of CdS followed by Cu–Cu_2_O nanorods on CC forms a CdS/Cu–Cu_2_O p–n heterojunction photoanode, resulting in superior photoelectrochemical (PEC) water‐splitting performance [59, 200, 206, 207]. Meanwhile, copper foil acts as a conductive and self‐transforming substrate that can evolve into Cu_3_Se_2_ during controlled growth, ensuring strong interfacial bonding and enhanced durability for efficient water splitting, as illustrated in Figure 4c [201, 208, 209].

Within this substrate framework, morphological and structural optimization emerges as a key strategy for enhancing performance. Hierarchically structured porous materials combining micro‐, meso‐, and macropores have been shown to facilitate electrolyte infiltration, ion diffusion, and electron transport, thereby maximizing the number of accessible catalytic sites [59, 80, 210, 211, 212]. As illustrated in Figure 4d, controlled nanoporosity in NiO transitioning from micro‐ to mesoporous architectures improved electrolyte diffusion and yielded an *η* of ~ 335 mV @ 10 mA cm^−2^ for OER [202]. Similarly, heteroatom doping (N, S, O) effectively tunes the local electronic structure, enhancing charge redistribution and proton adsorption/desorption kinetics during HER [213, 214]. For instance, Atchudan et al. synthesized a cashew‐nut‐skin‐derived heteroatom‐doped porous carbon (H‐PCM) exhibiting sponge‐like morphology and multifunctional dopants, achieving a Tafel slope of 75 mV dec^−1^ in 0.5 M H_2_SO_4_ [45]. Likewise, as shown in Figure 4e, NH_3_/H_2_O treatment followed by pyrolysis transforms precursor foams into nitrogen‐doped carbon frameworks with interconnected porosity, enhancing electron transport and exposing abundant active sites for water‐splitting [203]. Furthermore, Liu et al. demonstrated FeCoV‐NiMOF ultrathin nanosheet arrays (~1.5 nm) forming a 3D network with highly exposed surfaces, achieving efficient electrocatalysis as shown in Figure 4f [204]. Collectively, rational design combining hierarchical porosity, defect engineering, and heteroatom doping leads to superior intrinsic activity and durability in both metallic and biomass‐derived substrates.

Despite these advancements, conventional substrates like NF may overshadow intrinsic catalyst activity, as their high conductivity can artificially reduce measured *η*. CC may exhibit reduced stability under harsh alkaline or acidic environments, while copper‐based substrates are prone to corrosion unless suitably protected. Similarly, biomass‐derived carbons often suffer from low conductivity and dopant leaching under prolonged operation [67, 215]. Future research should thus focus on corrosion‐resistant hybrid scaffolds integrating conductive carbons with metallic backbones, controlled pore‐hierarchy engineering for balanced mass transport and site density, and standardized substrate benchmarking to decouple intrinsic catalyst behavior. The development of binder‐free, self‐supported, and recyclable electrodes compatible with biowaste‐derived catalysts will be critical for advancing sustainable, circular‐bio‐economy hydrogen production systems [210, 212, 214]. Such architectures uniquely leverage the dual role of biowaste‐derived carbons as both active catalytic hosts and lightweight conductive scaffolds, reducing the need for expensive metallic current collectors and further strengthening their techno‐economic advantage over conventional substrates.

## 5 Biowaste‐Derived Electrocatalysts for Water Splitting

### 5.1 HER Catalysts

HER is a key half‐reaction in EWS, where hydrogen ions are reduced to hydrogen gas through multi‐electron transfer at the cathode. It plays a vital role in sustainable hydrogen production for clean energy systems. Efficient HER requires catalysts that minimize *η* and accelerate reaction kinetics to achieve high current density with low energy input. While platinum‐based catalysts exhibit superior activity, their high cost and scarcity drive the development of alternative, earth‐abundant materials. Table 2 provides a comparison of current HER catalysts evaluated for activity, stability, and cost‐effectiveness.

**TABLE 2:** Biowaste‐derived catalysts for HER.

Biowaste Source	Catalyst Composition	Electrolyte	*η*_10_, mV	Tafel Slope, mV dec^−1^	Ref.
Rice husks	Corrugated graphene nanosheets	0.5 M H_2_SO_4_	9	31	[149]
Tamarind shells	Graphitic carbon	1.0 M KOH	221	204	[216]
Peanut shells	N‐doped carbon nanosheets	0.5 M H_2_SO_4_	390	75.7	[217]
Animal bones	N‐, P‐, Ca co‐doped biochar	0.5 M H_2_SO_4_	162 ± 3	80	[218]
Cattail fibers	Porous N‐doped carbon fibers	0.5 M H_2_SO_4_	248	135	[219]
Bean sprouts	N‐doped carbon	0.5 M H_2_SO_4_	413	98	[220]
Waste‐yeast cells	N, P co‐doped Mo_2_C in porous carbon	1.0 M KOH	84	58.15	[221]
Watermelon peels	Mo_2_C/C composite	1.0 M KOH	133	71	[222]
Walnut shells	Mo_2_C@C	0.5 M H_2_SO_4_	140	63	[90]
Sugarcane bagasse	Co–MoS_2_/C	0.5 M H_2_SO_4_	62	53.86	[223]
Banana waste	Pd/Fe_3_O_4_@carbon	0.5 M H_2_SO_4_	293	227.05	[224]
Chlorella	Co_2_P/N‐doped carbon	0.5 M H_2_SO_4_	151	50.21	[78]
Aloe waste	ZnMoO_4_/carbon	1.0 M KOH	124	54	[225]
Wood residue	Mo_2_C	0.5 M H_2_SO_4_	35	25	[226]

#### 5.1.1 Biowaste‐Derived Carbon‐Based Catalysts for HER

**FIGURE 5:**
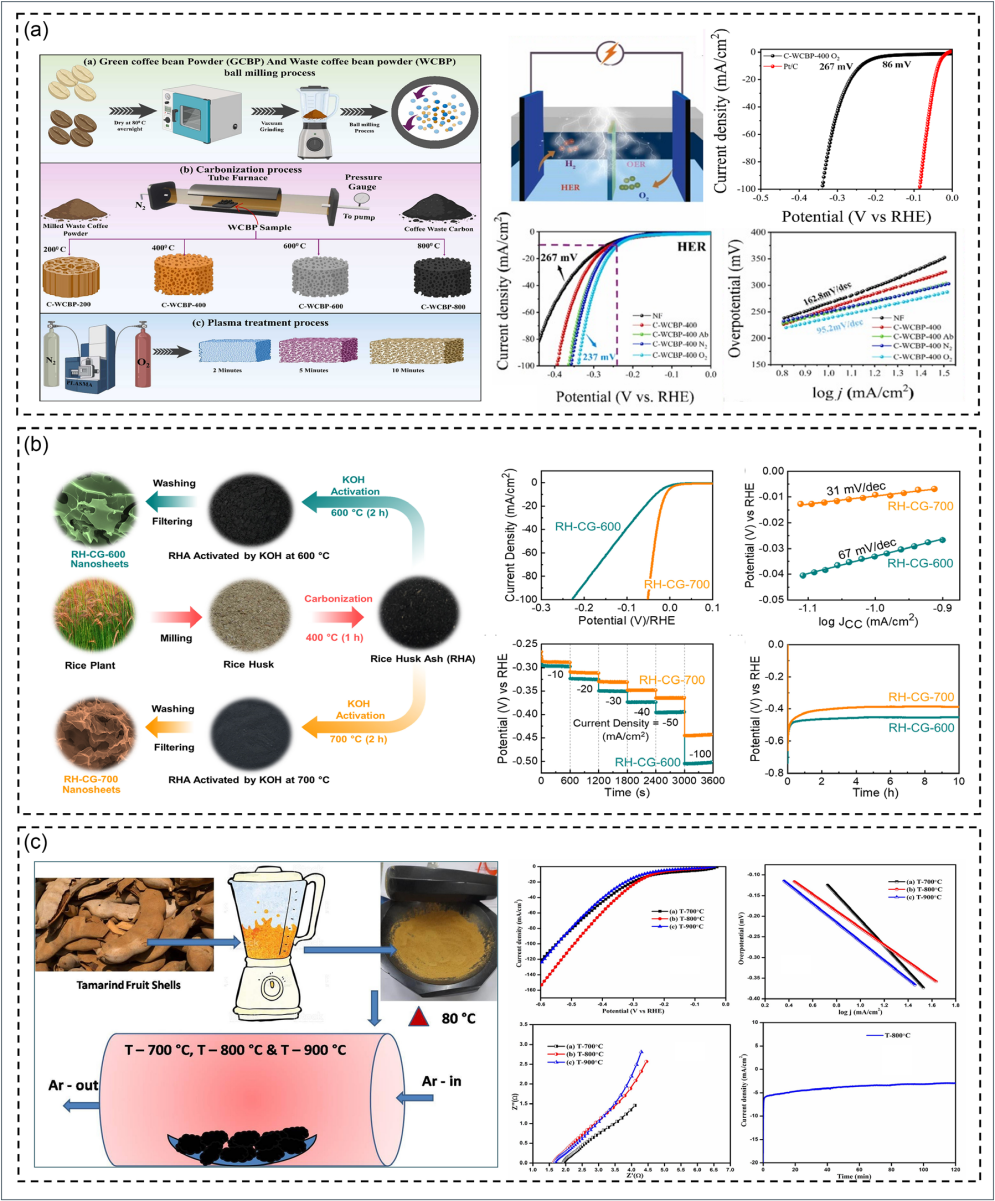
Biowaste‐derived carbon‐based catalysts for the HER: (a) coffee‐waste‐derived carbons activated via O_2_ plasma treatment showing enhanced HER/OER activity due to oxygen functionalization and improved graphitization. Reproduced with permission [74]. Copyright 2025, Elsevier. (b) Rice‐husk‐derived corrugated graphene nanosheets demonstrate temperature‐dependent HER performance and charge–transfer kinetics. Reproduced with permission [149]. Copyright 2022, Elsevier. (c) tamarind‐shell‐derived carbons synthesized at varied pyrolysis temperatures, highlighting optimal HER activity and stability at 800°C. Reproduced with permission [216]. Copyright 2022, Elsevier.

In recent years, biowaste‐derived carbon materials have emerged as sustainable and metal‐free catalysts for the HER, combining high electrical conductivity, tunable porosity, and abundant defect sites that enable the replacement of noble‐metal catalysts in water electrolysis. Among them, coffee‐waste‐derived carbon catalysts (Figure 5a demonstrated outstanding bifunctional activity after O_2_ plasma treatment, where the optimized C‐WCBP‐400‐O_2_ sample achieved a low HER *η* of 237 mV and Tafel slope of 95.2 mV dec^−1^. The superior performance was attributed to the plasma‐induced surface roughness, increased graphitic order, and oxygen‐functional groups, which enhanced charge transfer and intermediate adsorption during overall water splitting (OWS) [74]. Parallelly, rice‐husk‐derived corrugated graphene nanosheets (RH‐CG) prepared through KOH activation of rice‐husk ash have demonstrated exceptional electrocatalytic performance. As shown in Figure 5b, the RH‐CG‐700 electrode exhibited an ultralow *η* of 9 mV and a Tafel slope of 31 mV dec^−1^ in 0.5 M H_2_SO_4_, outperforming the RH‐CG‐600 counterpart due to its highly graphitized and mesoporous architecture, which enhances proton adsorption and accelerates charge transfer [149]. Likewise, peanut‐shell‐derived N‐doped carbons showed low onset potential (~0.08 V vs. RHE) and superior durability through heteroatom self‐doping and defect‐enriched graphitic layers [217]. Similarly, Tamarind‐shell‐derived carbon catalysts, prepared by direct pyrolysis using the tubular furnace method, exhibited remarkable electrocatalytic activity for the HER. As shown in Figure 5c, the optimized T‐800°C sample delivered a low *η* of 221 mV and a Tafel slope of 204 mV dec^−1^, demonstrating efficient catalytic kinetics. The superior performance was attributed to the formation of stable graphitic domains that enhanced electrical conductivity and facilitated effective hydrogen adsorption and desorption during electrolysis [216]. Further, animal‐bone‐derived N‐, P‐, Ca‐codoped biochar delivered *η* of 162 mV and 80 mV dec^−1^ slope due to synergistic heteroatom doping and optimized charge distribution [218]. Cattail‐fiber‐derived N‐doped porous carbon fibers provided 70 mV onset *η* and 135 mV dec^−1^ slope with > 97% current retention after 20 h, benefiting from microporous fibrous structure and strong conductivity [219]. In comparison, bean‐sprout‐derived N‐self‐doped carbon achieved *η* of 413 mV and 98 mV dec^−1^ slope through intrinsic N and O functional sites supporting efficient proton reduction [220].

Together, these studies illustrate that engineered biocarbon frameworks integrating heteroatom doping and graphitic ordering can deliver competitive HER activity within a circular bio‐economy context. Enhanced performance arises from heteroatom‐induced electronic modulation, which optimizes hydrogen adsorption, and improved graphitization that facilitates rapid electron transport. Comparative analysis indicates that highly graphitized and porous carbons exhibit superior activity due to better conductivity and active‐site accessibility, whereas poorly structured carbons show higher *η* [227, 228]. Despite notable progress, biowaste‐derived carbon catalysts for HER still suffer from nonuniform active sites, limited conductivity, and stability issues [229, 230]. Future efforts should emphasize controlled heteroatom doping, defect engineering, and in situ studies to optimize structure–activity relationships and enable scalable, durable catalysts for sustainable hydrogen production.

#### 5.1.2 Biowaste‐Derived Transition Metal‐Based Catalysts for HER

**FIGURE 6:**
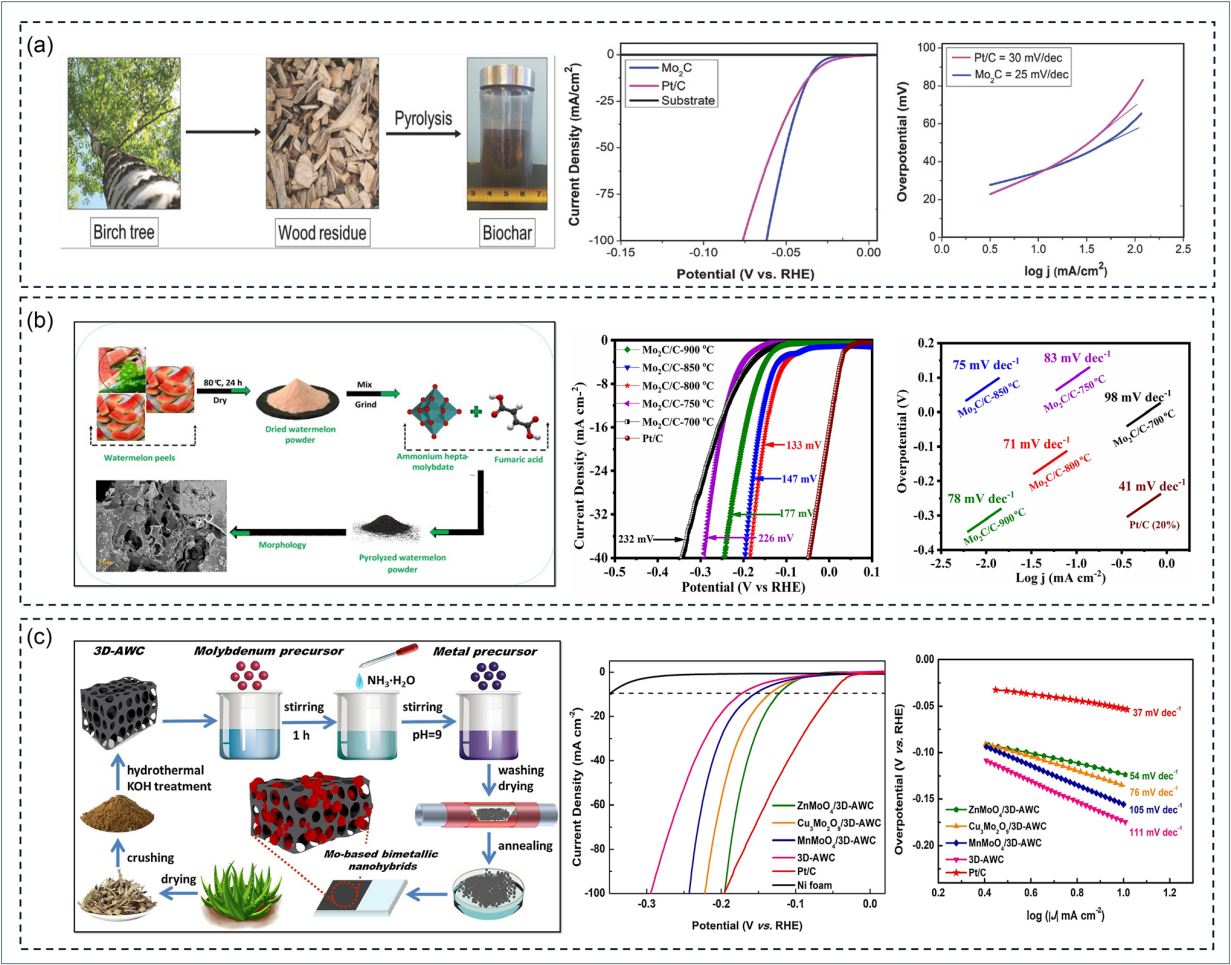
Biowaste‐derived transition‐metal‐based catalysts for the HER (a) biochar‐derived Mo_2_C exhibiting Pt‐like HER activity with low *η* and Tafel slope. Reproduced with permission [226]. Copyright 2018, Wiley. (b) watermelon‐rind‐derived Mo_2_C/C nanostructures showing temperature‐dependent catalytic performance and enhanced charge transfer. Reproduced with permission [222]. Copyright 2021, Elsevier. (c) 3D aloe‐waste‐derived carbon‐supported Mo‐based bimetallic oxides demonstrating synergistic metal–support interaction and superior HER activity in alkaline media. Reproduced with permission [225]. Copyright 2020, Elsevier.

Transition‐metal‐based electrocatalysts derived from biowaste have emerged as promising candidates to replace noble metals in the HER, combining high activity, cost‐effectiveness, and eco‐sustainability. The integration of transition metals (Mo, Co, Ni, Fe, Pd, etc.) into biomass‐derived carbon frameworks enhances electron transfer, optimizes hydrogen adsorption free energy, and stabilizes catalytic interfaces under electrochemical conditions. For instance, Yu et al. developed waste‐yeast‐derived N, P‐Mo_2_C nanoparticles confined in porous carbon (N, P‐Mo_2_C/NPC), achieving a low *η* of 84 mV and Tafel slope of 42 mV dec^−1^ in alkaline medium. The superior activity was attributed to N, P co‐doping that tuned the electronic density of Mo sites, optimizing H* adsorption and boosting catalytic kinetics through synergistic coupling between dopants and the carbon matrix [221]. As shown in Figure 6a, Humagain et al. reported biochar‐derived Mo_2_C nanostructures exhibiting remarkable Pt‐like HER activity with *η* of 35 mV at 10 mA cm^−2^ and a Tafel slope of 25 mV dec^−1^ in 0.5 M H_2_SO_4_, maintaining excellent stability beyond 100 h. The magnesiothermic solid‐state synthesis produced a porous Mo_2_C framework with a high surface area and low charge–transfer resistance, underscoring the potential of biochar as a scalable carbon precursor for efficient hydrogen evolution [226]. In another report, Yan et al. prepared porous β‐Mo_2_C@C clusters from walnut‐shell‐derived carbon, which achieved *η* of 140 mV at 10 mA cm^−2^ and Tafel slope of 63 mV dec^−1^. The porous structure facilitated electrolyte permeation and rapid hydrogen desorption, while the carbon matrix improved electrical conductivity and structural integrity during cycling [90]. Alongside it, biochar‐derived Mo_2_C/C catalysts fabricated from watermelon rind biomass showed excellent hydrogen evolution activity, as shown in Figure 6b. The optimized Mo_2_C/C‐800°C electrode delivered a low *η* of 133 mV at 10 mA cm^−2^ and a Tafel slope of 71 mV dec^−1^, outperforming samples prepared at other temperatures. This superior performance was attributed to its high surface area, enhanced conductivity, and stable Mo–C interface, enabling efficient and durable HER activity in alkaline media [222].

Furthermore, Ji et al. integrated Co‐doped MoS_2_ with sugarcane bagasse carbon (Co‐MoS_2_‐SCBC), achieving *η* of 62 mV and Tafel slope of 53.86 mV dec^−1^ in acidic medium. The Co atoms effectively substituted Mo sites, generating sulfur vacancies and facilitating metallic 1T‐phase transformation that improved conductivity and hydrogen adsorption kinetics [223]. Similarly, as shown in Figure 6c, Han et al. developed 3D aloe‐waste‐derived carbon‐supported Mo‐based bimetallic oxides (ZnMoO_4_/3D‐AWC) that delivered a low *η* of 124 mV and a Tafel slope of 54 mV dec^−1^, surpassing Pt/C in 1.0 M KOH. The outstanding performance was attributed to the enhanced electrical conductivity of the 3D carbon framework and the synergistic interaction between Zn and Mo, which facilitated efficient charge transfer and improved hydrogen evolution stability [225]. Kandathil et al. reported a Pd/Fe_3_O_4_@C nanocatalyst synthesized from banana pseudostem‐derived cellulosic carbon, delivering an *η* of 293 mV at 10 mA cm^−2^ in 0.5 M H_2_SO_4_. The magnetic carbon matrix enhanced recyclability and charge mobility while reducing noble metal loading [224].

Collectively, these biowaste‐derived transition‐metal‐based catalysts spanning carbides, sulfides, phosphides, and bimetallic oxides demonstrate that coupling heteroatom‐doped carbon frameworks with transition‐metal active sites yields superior HER performance [6, 59, 80, 231]. The synergy between metal–support interactions, defect engineering, and biogenic carbon architecture promotes efficient hydrogen evolution while aligning with circular bioeconomy principles [6]. However, limitations such as uncontrolled phase composition, nonuniform metal dispersion, and variability in biowaste precursors can lead to inconsistent catalytic performance and reduced long‐term stability. Future research should prioritize controlled heteroatom incorporation, phase engineering (1T vs. 2H transition‐metal dichalcogenides), and scale‐up of bioresource conversion pathways to advance sustainable hydrogen production [232, 233].

### 5.2 OER Catalysts

The OER is the anodic half of water splitting, where water molecules are oxidized to generate oxygen through a four‐electron transfer process. It is kinetically sluggish and requires a significant *η*, making it the rate‐limiting step in overall water electrolysis [196, 234, 235, 236]. Efficient OER catalysts are designed to lower this energy barrier and enhance reaction kinetics. Table 3 provides a comparison of current OER catalysts based on activity, stability, and *η* performance.

**TABLE 3:** Biowaste‐derived catalysts for OER.

Biowaste Source	Catalyst System	Electrolyte	***η***_**10**_, **mV**	**Tafel Slope, mV dec** ^**−1**^	Ref.
Lignosulfonate + Alginate (double biomass precursors)	Ru/RuS_2_‐Biochar Aerogel (Ru/RuS_2_‐BA‐900@WC)	0.5 M H_**2**_SO_4_	228	40	[237]
Watermelon peel	CoO@P‐doped biochar (CCW‐700)	1.0 M KOH	237	69.8	[164]
Ananas comosus leaves	CoMoO_4_/graphitized porous leaf carbon (CoMoO_4_/GPLC)	1.0 M KOH	289	60.4	[71]
Hazelnut Shell	Mo/Co supported on N‐doped biochar (BC‐Mo/Co)	1.0 M KOH	370	59	[238]
Cornstalk	Co, Fe,B, N, co‐doped biochar	1.0 M KOH	383	100.92	[239]
Onion peels	Fe_3_C@N‐doped carbon	1.0 M KOH	330	52	[240]
Banana peels	Ba_0.5_Sr_0.5_Co_0.8_Fe_0.2_O_3−δ_/Ndoped carbon	0.1 M KOH	350	65	[241]
Mangosteen skin	NiFe‐borate LDH/Ndoped carbon	1.0 M KOH	243	42.7	[242]
Milk powder	NiFeO_*x*_/N, P codoped carbon	1.0 M KOH	320	59.03	[243]
Human hair	NiO/C	1.0 M KOH	320	49	[244]

#### 5.2.1 Biowaste‐Derived Carbon‐Based Catalysts for OER

**FIGURE 7:**
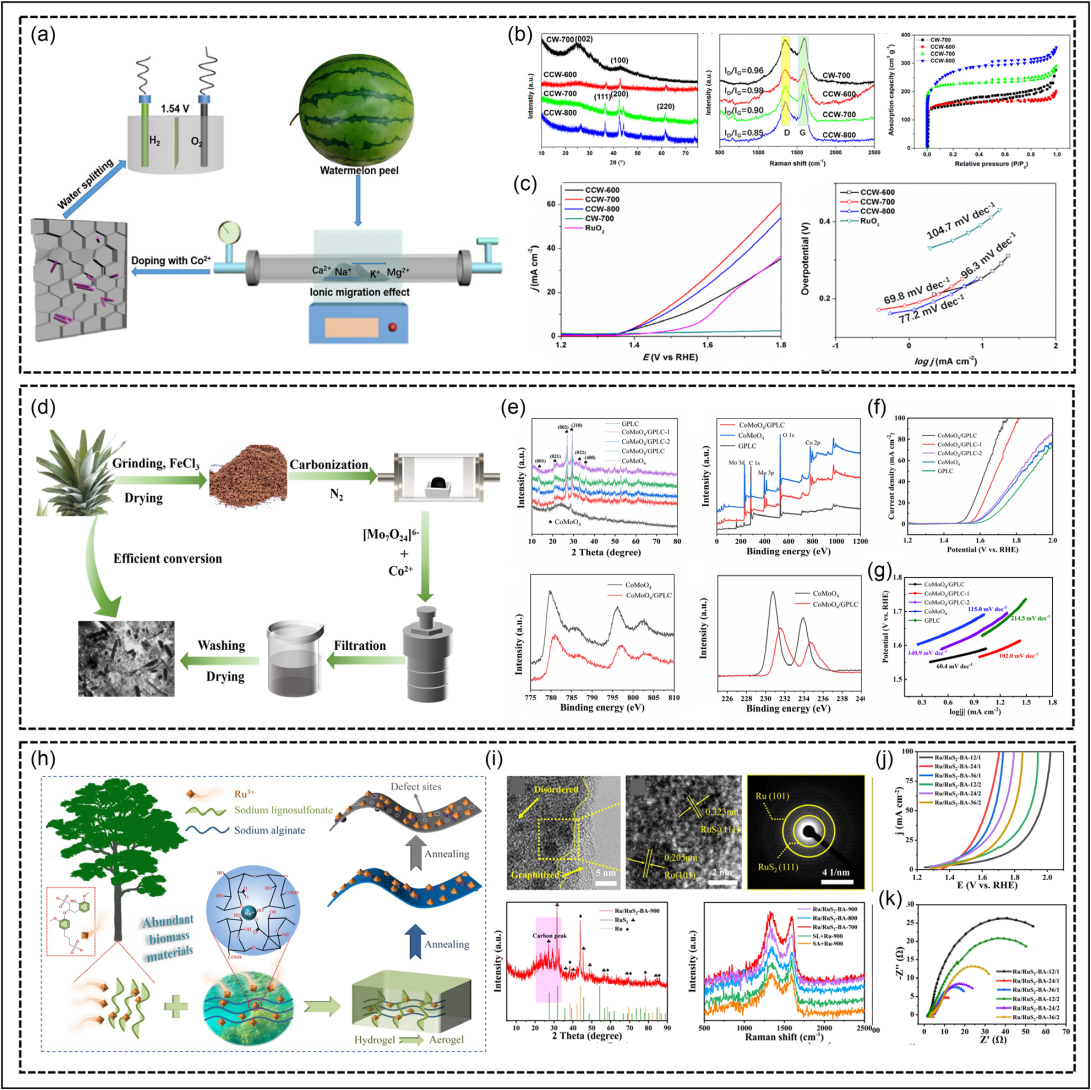
Biowaste‐derived carbon‐based catalysts for OER. (a–c) Synthesis, structural characterization, and OER performance of watermelon‐peel‐derived CCW‐700 catalyst [164]. Copyright 2021, ACS publication. (d–g) Fabrication, phase/surface analysis, and electrocatalytic performance of CoMoO_4_ nanorods grown on pineapple‐leaf‐derived GPLC [71]. Copyright 2024, MDPI. (h–k) Preparation, structural characterization, and OER activity, including impedance analysis of lignin/alginate‐derived Ru/RuS_2_ biochar aerogel [237]. Copyright 2022, Springer Nature.

The valorization of biomass into functional carbon electrocatalysts has emerged as a promising and sustainable strategy to simultaneously address environmental waste management and high‐performance electrochemical energy conversion. Biowaste‐derived carbons typically possess hierarchically porous structures, heteroatom doping (N, P, S), and active defect sites, which collectively promote fast charge transport, facilitate adsorption/desorption of oxygen intermediates, and stabilize metallic redox centers during oxygen evolution. Three representative examples, based on watermelon peel, pineapple leaf, and lignin/alginate‐derived aerogels, demonstrate how careful structural engineering and metal–carbon interfacial modulation yield high OER efficiency in both alkaline and acidic environments. Zhou et al. utilized discarded watermelon peels as a natural carbon precursor rich in alkali and alkaline‐earth ions, which induce in situ activation and pore formation during carbonization. The conversion of watermelon peel into a functional electrocatalyst demonstrates how naturally occurring minerals in biomass can assist in forming catalytically active porous carbons for OER. As illustrated in Figure 7a, discarded watermelon peels were carbonized and subjected to KOH activation, during which the intrinsic alkali and alkaline‐earth ions (Ca^2+^, Na^+^, K^+^, Mg^2+^) migrated and facilitated the formation of hierarchical porosity. Following activation, Co^2+^ ions were hydrothermally anchored onto the carbon support to generate uniformly dispersed CoO nanoparticles, forming the CCW‐700 composite. The structural properties are shown in Figure 7b, where XRD confirms the crystalline CoO phase and Raman spectra display an ID/IG ratio of ~0.90, indicating a defect‐rich carbon matrix with partial graphitization. Nitrogen sorption isotherms further reveal significant surface area enhancement, supporting effective electrolyte penetration during catalysis [164]. OER performance is shown in Figure 7c, where CCW‐700 exhibits a notably low *η* of 237 mV at 10 mA cm^−2^ and a Tafel slope of 69.8 mV dec^−1^ in 1 M KOH, outperforming commercial RuO_2_. This high catalytic activity arises from the synergistic interaction between the Co^2+^/Co^3^^+^ surface redox couple and the defect‐rich carbon backbone, which enhances charge transfer kinetics and stabilizes adsorbed *OH/*O/*OOH intermediates. The porous carbon network accelerates ion diffusion and oxygen bubble release, reducing kinetic resistance during operation. Moreover, CCW‐700 maintains excellent stability over prolonged electrolysis, emphasizing the strong carbon‐metal interfacial bonding. Overall, this system highlights how biomass‐activated carbon and in situ metal oxide formation together create efficient, low‐cost alkaline OER catalysts [164].

Wang et al. fabricated a high‐efficiency OER catalyst by growing CoMoO_4_ nanorods in situ on graphitized porous leaf carbon (GPLC) derived from waste Ananas comosus leaves. Figure 7 demonstrates the synthesis pathway and structural characterization of the biomass‐derived catalyst. In this work, waste pineapple leaves were first dried, ground, and mixed with iron salt, followed by carbonization under a nitrogen atmosphere to generate GPLC. The iron salt acted as a graphitization catalyst, improving the electrical conductivity of the biomass‐derived carbon. Afterward, cobalt and molybdenum precursors were introduced to grow CoMoO_4_ nanorods directly on the porous carbon matrix, forming the CoMoO_4_/GPLC composite as shown in Figure 7d. The porous carbon support provides a large surface area, abundant active sites, and rapid pathways for electron transfer, which are highly beneficial for OER performance [71]. Figure 7e further confirms the successful formation of the CoMoO_4_/GPLC catalyst through XRD and XPS analysis. The XRD patterns indicate the coexistence of crystalline CoMoO_4_ and graphitized carbon without impurity phases, suggesting that the biomass‐derived carbon support does not disturb the crystal growth of cobalt molybdate. XPS spectra reveal the presence of Co^2^^+^/Co^3^^+^ and Mo^6^^+^/Mo^4^^+^ species, which are important for redox reactions during OER. The emergence of mixed‐valence states suggests strong electronic interactions between CoMoO_4_ and the graphitized biomass carbon. Such interfacial charge transfer improves the adsorption and desorption of oxygen‐containing intermediates, thereby enhancing the intrinsic catalytic activity [71]. The electrochemical performance shown in Figure 7f,g proves that the biomass‐derived carbon support significantly improves OER activity. The CoMoO_4_/GPLC catalyst exhibited a low *η* of about 289 mV at 10 mA cm^−2^ and a relatively small Tafel slope of around 60.4 mV dec^−1^, indicating faster reaction kinetics compared with pristine CoMoO_4_ and other control samples. The enhanced activity can be attributed to the synergistic effect between porous graphitized carbon and CoMoO_4_ nanorods. The biomass‐derived carbon matrix not only increases conductivity but also facilitates electrolyte penetration, charge transport, and oxygen bubble release. Therefore, biowaste‐derived carbon materials can serve as highly effective support for OER catalysts, offering a sustainable and low‐cost strategy for high‐performance electrocatalysis [71].

Furthermore, Hui et al. developed a biochar‐aerogel‐based Ru/RuS_2_ catalyst by co‐utilizing terrestrial lignosulfonate and marine alginate precursors. Figure 7h illustrates the preparation strategy of the biomass‐derived RuNi aerogel catalyst using abundant natural wood resources. Sodium lignosulfonate and sodium borate were employed as the main precursors to construct a three‐dimensional hydrogel network, followed by annealing treatment to form a porous aerogel framework embedded with Ru and Ni species. The use of biomass‐derived lignin is particularly important because it provides a sustainable carbon source with abundant oxygen‐containing functional groups, which can facilitate the uniform distribution of active metal sites. The final RuNi aerogel exhibits a highly porous interconnected structure, which is beneficial for mass transport, electrolyte diffusion, and exposure of catalytic active sites during the OER. Figure 7i,j presents the structural and compositional characterization of the RuNi aerogel catalyst. TEM analysis reveals that ultrafine Ru species are uniformly dispersed within the Ni‐based matrix without obvious agglomeration. The high‐resolution TEM image confirms the presence of well‐defined lattice fringes corresponding to metallic Ni and Ru‐containing phases, indicating strong interaction between the two metals. The XRD pattern demonstrates the crystalline nature of the RuNi alloy framework, while Raman spectra indicate the presence of disordered carbon and defect‐rich structures generated from the biomass precursor. These defect sites can improve electron transport and provide additional adsorption centers for OER intermediates. Furthermore, the elemental mapping confirms the homogeneous distribution of Ru, Ni, carbon, and oxygen throughout the aerogel structure, suggesting successful integration of all active components [237].

The electrochemical results shown in Figure 7j,k confirm the superior OER performance of the biomass‐derived RuNi aerogel catalyst. The polarization curves indicate that the optimized RuNi aerogel requires a lower *η* to achieve high current densities compared with the control samples. In addition, the Tafel slope is significantly reduced, demonstrating faster reaction kinetics and more efficient charge–transfer behavior. The improved catalytic activity can be attributed to the synergistic effect between the porous biomass‐derived carbon network and the RuNi active sites. The hierarchical porous structure enhances electrolyte penetration and gas release, while the defect‐rich carbon matrix improves conductivity and stabilizes the metal nanoparticles. As a result, biomass‐derived RuNi aerogels represent a promising class of high‐performance OER catalysts with excellent activity and structural stability [237]. Moreover, interface‐controlled synthesis strategies are required to strengthen the bonding between carbon frameworks and transition‐metal active phases, ensuring long‐term catalytic durability under industrial current densities. The expansion to acidic and neutral media remains a critical challenge, necessitating stabilization mechanisms such as carbon‐anchored noble‐metal sulfides, phosphides, or single‐atom active centers. In addition, developing predictive computational‐experimental design platforms will allow rational screening of waste precursors and process conditions rather than empirical trial‐based optimization. From a practical standpoint, attention must also be given to scalable, low‐emission carbonization and activation processes, along with life‐cycle sustainability assessments to validate environmental benefits. Integration of biowaste‐derived catalysts into membrane electrolyzer systems and hybrid or tandem PEC OER configurations represents a key step toward real‐world deployment. Collectively, these directions will advance biowaste‐based catalysts from laboratory success to viable green hydrogen manufacturing platforms.

The enhanced OER performance primarily arises from the synergistic effects of hierarchical 2D/3D porous architectures, defect‐rich carbon matrices, and strong interfacial coupling between active species and the carbon host [6, 80, 245, 246, 247]. These features facilitate rapid mass transport, increase active site exposure, and optimize electronic structures, thereby improving adsorption/desorption kinetics and charge transfer. However, challenges remain, including limited control over defect density, aggregation and dissolution of active metal species under acidic conditions, and poor reproducibility due to the inherent heterogeneity of biowaste precursors. Additionally, the exact nature of active sites and dynamic structural evolution during operation is still not fully understood [81, 248]. Future efforts should focus on precise defect engineering, stabilization of active phases, and advanced in situ/operando studies combined with theoretical modeling to establish clear structure activity relationships and enable scalable, durable electrocatalyst design [249, 250, 251].

#### 5.2.2 Biowaste‐Derived Transition Metal‐Based Catalysts for OER

The conversion of biowaste into carbonaceous supports for transition metal‐based OER catalysts has emerged as an effective strategy for developing sustainable, low‐cost, and high‐performance electrocatalysts. Biowaste precursors naturally possess hierarchical architecture, oxygen‐containing functional groups, and abundant heteroatoms such as nitrogen, sulfur, and oxygen. Upon pyrolysis, these features can be transformed into porous graphitic carbon frameworks with large specific surface area, rich defect density, and high electrical conductivity. Such carbon matrices serve as ideal support for transition metal species because they promote strong metal anchoring, suppress nanoparticle agglomeration, and facilitate rapid electron transport during electrocatalysis. Furthermore, heteroatom doping can alter the electronic distribution of the carbon lattice, optimize the adsorption energy of oxygen‐containing intermediates (*OH, *O, and *OOH), and enhance the intrinsic OER activity.

**FIGURE 8:**
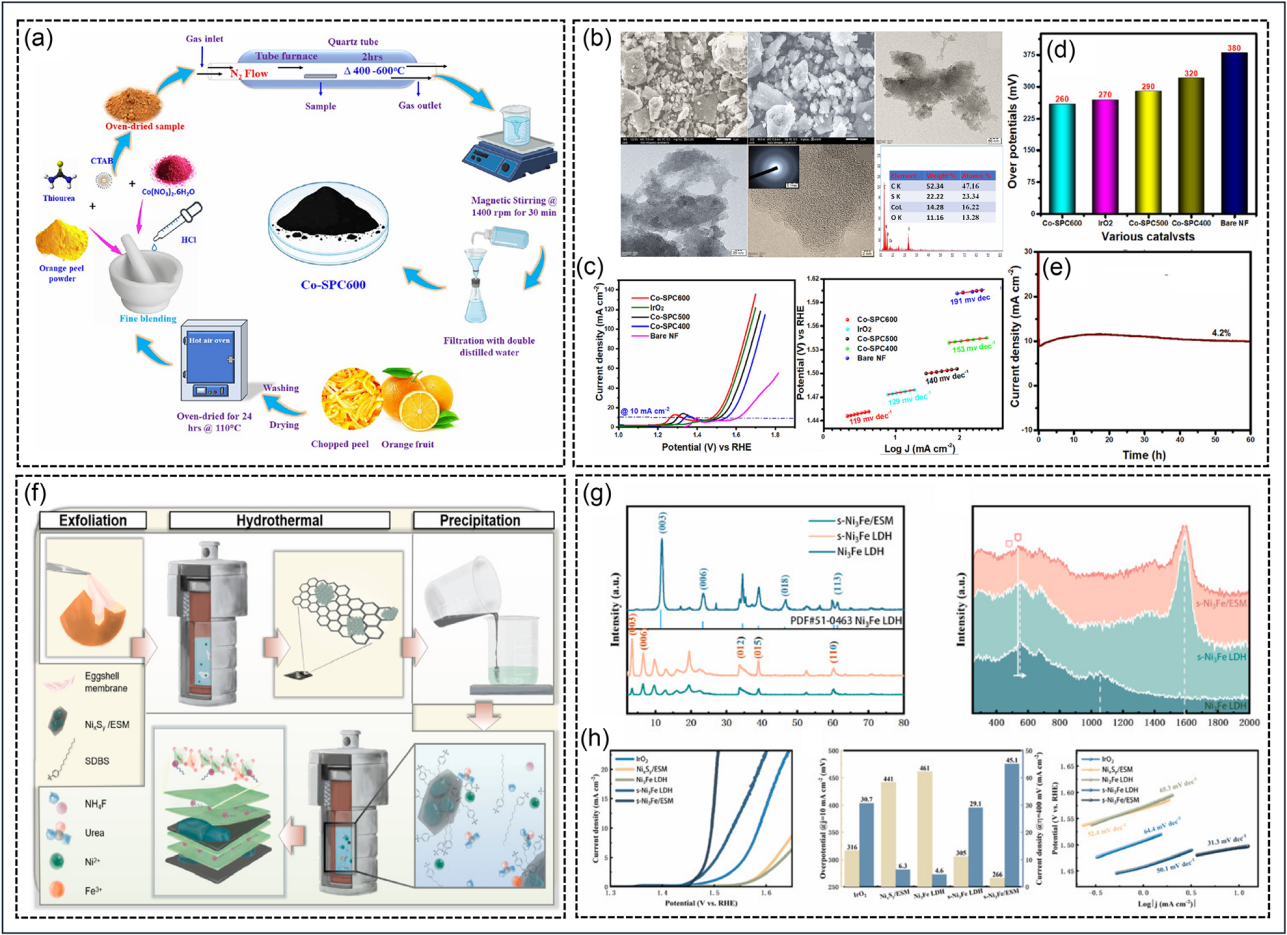
Biowaste‐derived transition metal carbon hybrid catalysts for OER (a–e) sulfur‐doped porous graphitic carbon‐supported cobalt catalyst (Co‐SPC600) derived from waste orange peel [86]. Copyright 2025, ACS Publication. (f–h) eggshell membrane (ESM)‐derived NiFe‐LDH system [252]. Copyright 2024, Elsevier.

A representative example is the sulfur‐doped porous graphitic carbon‐supported cobalt catalyst (Co‐SPC600) derived from waste orange peel, as shown in Figure 8a. In this synthesis route, orange peel waste was first washed, dried, and pulverized, followed by blending with cobalt nitrate, thiourea, and cetyltrimethylammonium bromide (CTAB). Thiourea served as the sulfur source, while CTAB promoted pore formation and uniform precursor dispersion. The resulting mixture was pyrolyzed at different temperatures under a nitrogen atmosphere, leading to the formation of sulfur‐doped graphitic carbon embedded with cobalt nanoparticles. During pyrolysis, the organic constituents of the orange peel decomposed into a porous carbon skeleton, whereas sulfur atoms were incorporated into the graphitic lattice, creating electron‐rich defect sites. Simultaneously, cobalt ions were converted into metallic cobalt nanoparticles and partially oxidized cobalt species, which became encapsulated within thin graphitic carbon shells. Such graphitic encapsulation is highly beneficial because it minimizes particle aggregation, prevents cobalt dissolution, and improves long‐term electrochemical stability [86].

The structural and morphological characterization results of Co‐SPC600 are presented in Figure 8b. SEM images reveal an interconnected porous carbon framework with irregular nanoscale cavities, indicating that the biomass‐derived matrix provides abundant exposed surface area and mass transport channels. TEM and HRTEM images further confirm that cobalt nanoparticles are uniformly dispersed within the graphitic carbon matrix and are wrapped by thin graphitic layers. The observed lattice fringes indicate the high crystallinity of the cobalt nanoparticles, while the surrounding graphitic shells establish continuous conductive pathways for electron transport. In addition, EDS analysis verifies the homogeneous distribution of Co, C, S, and O throughout the material, confirming the successful incorporation of sulfur dopants and cobalt active sites.

The OER activity of the Co‐SPC catalysts is shown in Figure 8c–e. Among the different carbonization temperatures, Co‐SPC600 exhibits the best performance, requiring *η* of approximately 260 mV to achieve a current density of 10 mA cm^−2^. This value is lower than those of Co‐SPC400, Co‐SPC500, and bare nickel foam, indicating that the optimized graphitization degree and defect concentration are achieved at 600°C. The Tafel slope of Co‐SPC600 is also significantly lower, suggesting faster OER kinetics and improved charge–transfer capability. Moreover, the long‐term chronoamperometric test reveals only a 4.2% loss in current density after prolonged operation, demonstrating excellent electrochemical durability. The superior catalytic activity can be attributed to the synergistic effects between sulfur‐doped graphitic carbon and cobalt nanoparticles. Sulfur doping modifies the local electron density of the carbon framework, cobalt nanoparticles provide highly active redox sites through Co^2^^+^/Co^3^^+^ transitions, and the porous graphitic network accelerates electron transport, electrolyte penetration, and oxygen bubble release [86].

Another notable example of biomass‐derived OER catalysts is the eggshell membrane (ESM)‐derived NiFe‐LDH system shown in Figure 8f–h. ESM is a naturally fibrous and protein‐rich biowaste material that contains abundant sulfur‐ and nitrogen‐containing functional groups. After exfoliation and hydrothermal treatment, the ESM is converted into a conductive carbonaceous scaffold containing sulfur‐rich Ni‐S domains. These domains subsequently act as nucleation centers for the in situ growth of NiFe‐LDH nanosheets. The incorporation of sodium dodecylbenzene sulfonate (SDBS) is particularly important because it enlarges the interlayer spacing of the LDH sheets, enhances nanosheet dispersion, and promotes exposure of catalytically active sites [252].

As shown in Figure 8g, XRD analysis confirms the successful formation of the NiFe‐LDH phase, while Raman spectroscopy indicates the coexistence of defect‐rich carbon and layered hydroxide structures. The broad D and G bands demonstrate the presence of disordered graphitic carbon, which can provide additional defect sites and improve conductivity. Morphological observations further reveal vertically aligned ultrathin LDH nanosheets strongly anchored onto the biomass‐derived carbon substrate. Such architecture is highly favorable for OER because it exposes more electrochemically active sites, shortens ion diffusion pathways, and facilitates efficient release of oxygen bubbles from the catalyst surface [252]. The electrochemical performance results shown in Figure 8h demonstrate that the s‐Ni3Fe/ESM catalyst delivers excellent OER activity, achieving *η* of approximately 266 mV at 10 mA cm^−2^, which is very close to that of commercial IrO_2_ catalysts. The Tafel slope is also considerably reduced compared with the undoped or unsupported samples, indicating accelerated reaction kinetics. In addition, impedance analysis confirms a lower charge–transfer resistance, suggesting that the intimate contact between the NiFe‐LDH nanosheets and the conductive carbon scaffold greatly improves electron transfer. The enhanced catalytic performance arises from the strong electronic coupling between NiFe‐LDH and the sulfur‐doped carbon framework, as well as the formation of NiOOH/FeOOH active species during OER operation.

Overall, these examples clearly demonstrate that biowaste‐derived carbon frameworks can significantly improve the activity and stability of transition metal‐based OER catalysts. The combination of hierarchical porosity, heteroatom doping, defect engineering, and strong metal–support interaction creates highly efficient catalytic systems with fast electron transport and optimized adsorption behavior [253, 254, 255, 256, 257]. Future research should focus on understanding the dynamic surface reconstruction of such catalysts under operational conditions through operando characterization techniques and density functional theory calculations. In addition, scalable biomass conversion methods and integration into practical alkaline or seawater electrolyzers will be essential for translating these sustainable catalyst systems into real‐world hydrogen production technologies [258, 259, 260, 261].

### 5.3 Comparative Analysis of HER versus OER Requirements

Although both the HER and OER are integral to EWS, the catalytic requirements for these two half‐reactions are fundamentally different. HER is a two‐electron cathodic reduction process, whereas OER is a four‐electron anodic oxidation process involving multiple proton‐coupled electron‐transfer steps and more complex surface intermediates. As a result, HER generally demands catalysts that provide rapid electron transport and optimized hydrogen adsorption energetics, while OER requires catalysts that can not only adsorb and transform oxygen‐containing intermediates efficiently, but also withstand harsh oxidative potentials over prolonged operation. This intrinsic kinetic and mechanistic asymmetry explains why HER and OER are rarely optimized by identical catalyst properties, even within the same biowaste‐derived material platform [251, 262, 263]. The key distinctions in reaction characteristics and catalyst design requirements are systematically compared in Table 4.

**TABLE 4:** Comparative analysis of HER versus OER Requirements.

Property	HER	OER
Fundamental process	2e^−^ cathodic reduction	4e^−^ anodic oxidation
Main mechanistic routes	Volmer–Heyrovsky/Volmer–Tafel	Adsorbate evolution mechanism, lattice‐oxygen mechanism
Key surface intermediates	H^*^	OH*, O*, OOH*
Dominant catalytic requirement	Fast charge transfer and optimized H adsorption/desorption	Balanced oxygen‐intermediate adsorption and strong oxidative stability
Most critical material property	Electrical conductivity	Structural/chemical durability under anodic bias
Typical effective catalyst classes	Graphitized carbons, heteroatom‐doped carbons, carbides, phosphides, sulfides, metal‐carbon hybrids	Oxides, oxyhydroxides, LDHs, spinels, metal oxide/carbon hybrids
Major role of biomass‐derived carbon	Active conductive framework and electronic modulator	Conductive scaffold/support for dispersed OER‐active phases
Main design challenge	Tuning H* binding while maintaining conductivity and active‐site accessibility	Stabilizing redox‐active metal centers and preventing corrosion/reconstruction‐induced deactivation

For HER, the essential requirement is the efficient adsorption and desorption of hydrogen intermediates (H*), ideally with near‐thermoneutral hydrogen binding. In acidic media, this descriptor is often closely linked to intrinsic catalytic activity, whereas in alkaline media, additional catalytic assistance is required for water dissociation before hydrogen adsorption can proceed. Therefore, conductive carbon frameworks, graphitized biocarbons, metal carbides, sulfides, phosphides, and metal‐carbon hybrids are particularly effective HER candidates because they combine high electrical conductivity with tunable hydrogen‐binding sites. This interpretation is consistent with the HER systems discussed in this review, where graphitized biocarbons, heteroatom‐doped carbons, and transition‐metal/biocarbon composites show improved HER performance through enhanced charge transfer, defect‐mediated active‐site generation, and optimized interfacial hydrogen adsorption. In this context, conductivity, graphitic ordering, and electronic modulation of the active center are especially critical for HER‐oriented catalyst design [263, 264, 265].

In contrast, OER places more stringent demands on catalyst stability and surface chemical adaptability. Because OER proceeds through oxygenated intermediates such as OH*, O*, and OOH*, catalyst surfaces must stabilize these species in a balanced manner without becoming over‐bound or undergoing rapid degradation. Moreover, the strongly anodic conditions of OER often induce surface reconstruction, oxidation, metal dissolution, carbon corrosion, or phase transformation, particularly under large current density operation. Accordingly, the most effective OER catalysts are often transition‐metal oxides, oxyhydroxides, LDHs, spinels, or oxide/hydroxide‐carbon hybrids, rather than purely conductive carbon materials alone. This distinction is also reflected in the present review's OER discussion, where biowaste‐derived carbon frameworks mainly act as conductive and porous scaffolds, while the true OER enhancement arises from interfacial coupling with catalytically active Co‐, Ni‐, Fe‐, Mo‐, or Ru‐based phases. Thus, for OER, durability under oxidative bias, retention of active structure, and stabilization of oxygen‐evolution intermediates are typically more decisive than conductivity alone [251, 262, 266].

From the perspective of biowaste‐derived electrocatalysts, this comparison highlights an important design principle: the same biomass precursor may not yield equally optimal HER and OER catalysts unless its structure is selectively engineered according to reaction‐specific demands. For HER, biomass‐derived carbons benefit from high graphitization, heteroatom doping, defect exposure, and intimate coupling with HER‐active phases that regulate hydrogen adsorption and accelerate charge transfer. For OER, however, the priority shifts toward stabilizing redox‐active metal centers, generating robust metal–oxygen coordination environments, and preserving catalytic integrity under oxidative polarization. Therefore, bifunctional biowaste‐derived catalysts should not be interpreted simply as materials that perform both reactions acceptably; rather, they should be understood as deliberately engineered systems that reconcile the conductivity‐driven requirements of HER with the stability‐ and intermediate‐management requirements of OER. This reaction‐specific interpretation provides a clearer framework for comparing catalyst performance across your HER and OER sections and directly addresses the need for a systematic distinction between the two reactions [79, 267, 268].

### 5.4 OWS Performance

OWS combines hydrogen and OERs to produce hydrogen fuel directly from water. It is a key step toward sustainable and carbon‐free energy conversion. Efficient OWS catalysts aim to minimize *η*, enhance charge transfer, and maintain stability under operation. Table 5 provides a comparison of current OWS performances based on activity, stability, and energy efficiency [284, 285, 286].

**TABLE 5:** Biowaste‐derived catalysts for OWS.

Biowaste Source	Catalyst Composition	Electrolyte	E_10_, V	Durability/Stability	Reference
Camellia flower	S‐doped carbon	1.0 M KOH	1.76	24 h @ ~20 mA cm^−2^	[269]
Corn stalks	Few‐layer N‐doped porous carbon	1.0 M KOH	1.65	30 h @ 1.65 V	[270]
Rose flower	Ni‐doped graphitic carbon	1.0 M KOH	1.64	24 h @ 1.64 V	[271]
Textile sludge	Fe, N co‐doped carbon	1.0 M KOH	1.64	14 h @ 1.7 V	[272]
Alfalfa	NiFe/N, P, S co‐doped carbon	1.0 M KOH	1.60	50 h @ 10 mA cm^−2^	[273]
Magnolia leaves	CoP/C	1.0 M KOH	1.56	24 h @ 1.59 V	[274]
Polysaccharide chitin	Co_2_P/N, P co‐doped carbon	1.0 M KOH	1.65	10 h @ ~20 mA cm^−2^	[275]
Ginkgo leaves	Co_2_P@CoP/C	1.0 M KOH	1.63	1000 min @ 10 mA cm^−2^	[276]
Cauliflower leaves	Ni/NiO/N‐doped carbon	0.1 M KOH	1.688	20 h @ 10–30 mA cm^−2^	[277]
Amaranth	Fe, N co‐doped carbon	1.0 M KOH	1.53	30 h @ 1.53 V	[278]
Lotus leaves	Co/MoO_2_@N‐doped carbon	1.0 M KOH	1.629	48 h @ 10 mA cm^−2^	[279]
Chicken feathers	Ni‐Co oxides/C	1.0 M KOH	1.53	200 h @ 1.7 V	[280]
Tissue paper	Co_9_S_8_@Co–N/C nanorods	1.0 M KOH	1.61	70 h @ 10 mA cm^−2^	[281]
Catkin	MoS_2_@NiOOH@C	1.0 M KOH	1.62	40 h @ 1.62 V	[282]
Willow catkins	NiFe LDH/(NiFe)S_*x*_ hollow carbon	1.0 M KOH	1.53	100 h @ 10 mA cm^−2^	[283]

#### 5.4.1 Biowaste‐Derived Metal‐Free Carbon Catalysts for OWS

**FIGURE 9:**
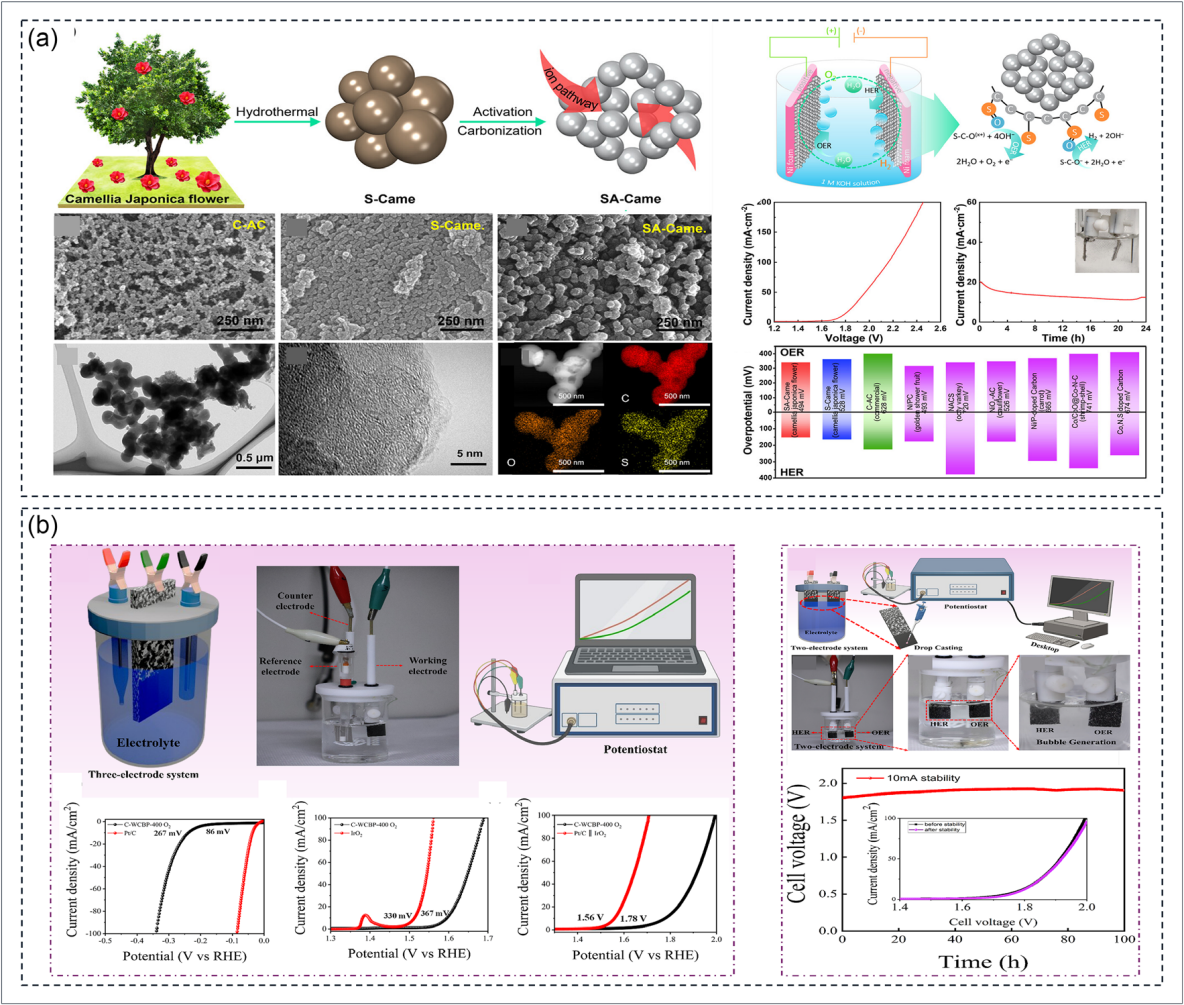
Biowaste‐derived metal‐free carbon catalysts for OWS (a) sulfur self‐doped camellia‐derived porous carbon exhibiting bifunctional activity with low *η* for HER and OER and stable performance over 24 h. Reproduced with permission [269]. Copyright 2022, Wiley. (b) plasma‐treated waste‐coffee‐derived carbon electrode showing efficient OWS at 1.78 V for 10 mA cm^−2^ with excellent durability and charge–transfer characteristics. Reproduced with permission [74]. Copyright 2025, Elsevier.

Biowaste‐derived carbon materials have emerged as bifunctional, metal‐free electrocatalysts for OWS, offering abundant heteroatom‐doped active sites, high electrical conductivity, and hierarchical porosity that enable simultaneous hydrogen and OERs. Such materials promote electron transfer and intermediate adsorption through synergistic structural and electronic modifications arising from their bio‐origin. Among biowaste‐derived metal‐free carbons for OWS, sulfur self‐doped activated camellia carbon (SA‐Came) is notable: Figure 9a shows a two‐electrode LSV in 1.0 M KOH reaching 10 mA cm^−2^ at 1.76 V, while the paired CA trace maintains stable current over 24 h; consistent half‐cell metrics for this metal‐free carbon include HER *η*_10_ ≈ 154 mV (Tafel ≈ 89.9 mV dec^−1^) and OER *η*_10_ ≈ 340–362 mV (Tafel ≈ 86 mV dec^−1^), attributable to the S‐doped porous carbon nanospheres that enhance conductivity, create polar active sites, and accelerate charge transfer across the carbon framework [269]. In a related study, Sreenivasulu et al. developed Ni‐incorporated N‐doped graphitic carbon from pomegranate peel waste, achieving a low overall splitting potential of 1.51 V at 10 mA cm^−2^ and excellent long‐term stability (>150 h). The improved performance arose from nitrogen‐induced defect states and graphitic ordering that facilitated both HER and OER pathways [85, 95]. As shown in Figure 9b, Ghode et al. reported that waste coffee‐derived carbon (C‐WCBP‐400‐O_2_), produced through plasma‐assisted surface modification, achieved a cell voltage of 1.78 V at 10 mA cm^−2^ in 1 M KOH, surpassing that of bare NF (1.86 V) [74]. The remarkable electrocatalytic activity was attributed to oxygen functionalization, which improved hydrophilicity, charge transport, and surface wettability, enabling stable operation for 100 h without noticeable degradation, confirming its durability for OWS. Similarly, Xing et al. synthesized β‐Mo_2_C nanoparticles embedded in cornstalk‐derived carbon nanosheets, yielding a cell voltage of 1.65 V at 10 mA cm^−2^ with negligible loss after 30 h operation, benefiting from the synergistic interaction between Mo_2_C domains and the conductive carbon matrix that promoted charge transfer and dual‐site reactivity [270]. Moreover, Jiang et al. fabricated biomass carbon tubes from cotton fibers decorated with metallic Co, achieving an impressively low overall water‐splitting voltage of 1.40 V at 10 mA cm^−2^, highlighting how the microtubular carbon structure accelerates ion diffusion and electron transport while maintaining mechanical robustness during long‐term operation [287].

Collectively, these studies underscore the potential of biowaste‐derived carbon architectures as eco‐sustainable and scalable electrocatalysts for OWS [276, 288]. Their performance originates from heteroatom‐induced charge redistribution, hierarchical porosity, and intrinsic carbon conductivity, positioning them as viable candidates for green hydrogen generation within a circular bio‐economy framework [289]. These effects arise from optimized intermediate adsorption and enhanced charge–transfer pathways enabled by heteroatom doping and graphitic structures. Compared to metal‐incorporated systems, metal‐free carbons often exhibit higher cell voltages due to lower intrinsic active‐site activity. However, challenges such as limited control over heteroatom configuration and variability in biowaste composition still affect reproducibility and performance consistency. Future work should focus on precise heteroatom engineering and advanced characterization to establish reliable structure–activity relationships [276, 288].

#### 5.4.2 Biowaste‐Derived Transition‐Metal‐Based Catalysts for OWS

**FIGURE 10:**
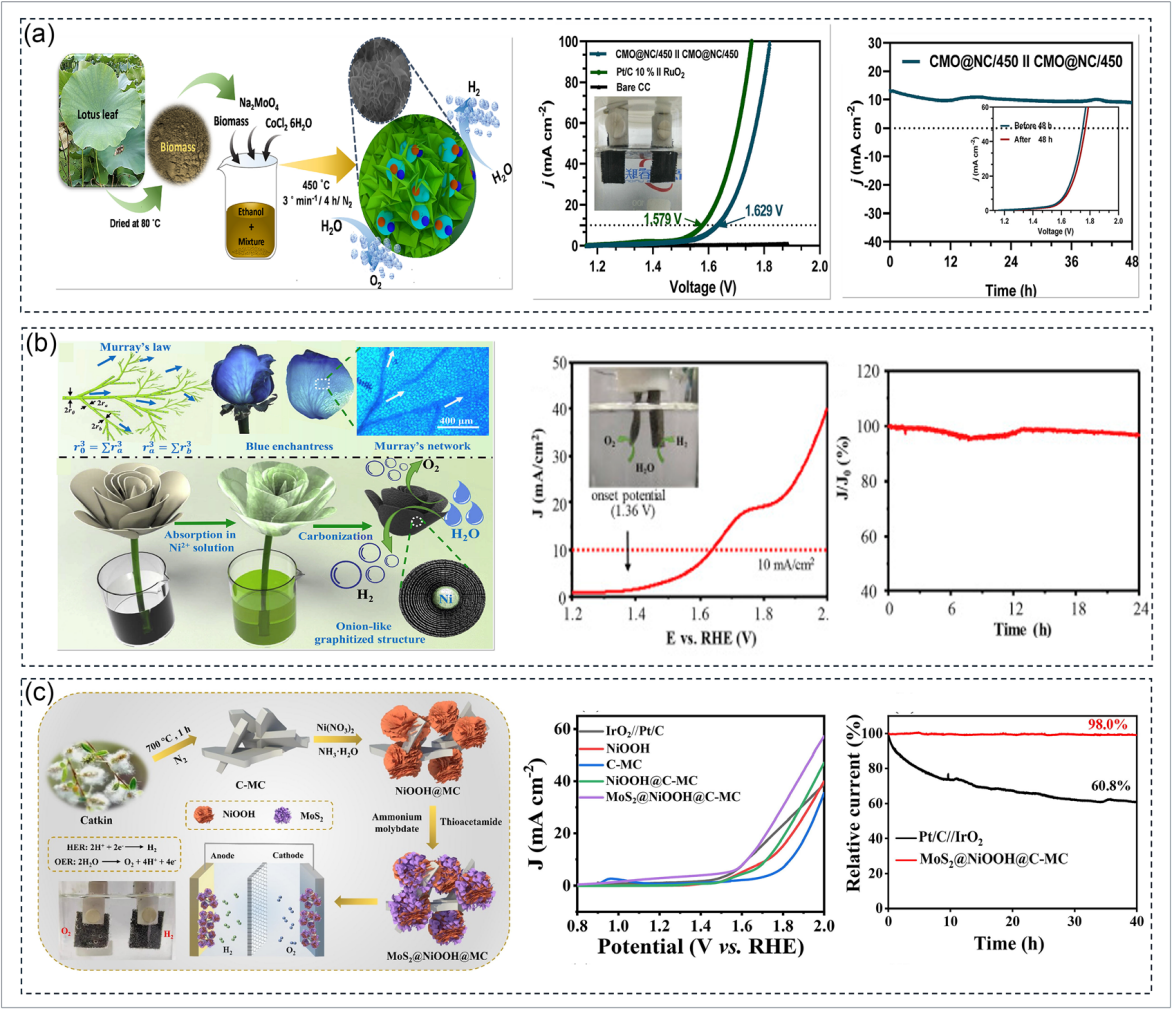
Biowaste‐derived transition‐metal‐based catalysts for OWS (a) lotus‐leaf‐derived Co/MoO_2_@N‐doped carbon nanosheets achieving 10 mA cm^−2^ at 1.629 V with excellent stability due to synergistic Co‐MoO_2_/N–C coupling. Reproduced with permission [279]. Copyright 2022, Elsevier. (b) rose‐petal‐derived Ni‐doped graphitic carbon exhibiting high graphitization and durable bifunctional activity through Ni‐C active‐site synergy. Reproduced with permission [271]. Copyright 2019, Elsevier. (c) willow‐catkin‐derived MoS_2_@NiOOH@C‐MC hybrid delivering 1.62 V at 10 mA cm^−2^ with 98% current retention, integrating HER‐active MoS_2_ and OER‐active NiOOH on a conductive carbon scaffold. Reproduced with permission [282]. Copyright 2022, Elsevier.

Transition‐metal‐based catalysts derived from biomass precursors have demonstrated significant potential as bifunctional electrodes for OWS, integrating abundant active sites, high electrical conductivity, and stability with sustainable biowaste valorization [290]. Such systems combine the redox flexibility of metals such as Co, Ni, Fe, Mo, and Cu with the porous and heteroatom‐doped carbon frameworks obtained from natural precursors, resulting in remarkable catalytic synergy. As shown in Figure 10a, lotus‐leaf‐derived Co/MoO_2_@N‐doped carbon nanosheets (CMO@NC/450) achieved 10 mA cm^−2^ at 1.629 V in 1.0 M KOH, closely matching Pt/C‖RuO_2_ (1.579 V) and maintaining stability for 48 h. The excellent bifunctional activity arises from the hierarchical nanosheet structure and Co‐MoO_2_/N‐doped carbon synergy, which enhances charge transfer and active‐site accessibility [279]. Similarly, Amiinu et al. converted waste cellulose tissue into Co_9_S_8_@Co–N/C nanorods, achieving a low overall cell voltage of 1.61 V at 10 mA cm^−2^ with durability over 70 h in 1 M KOH [281]. The synergistic coupling between Co_9_S_8_ and Co–N‐doped carbon enabled rapid charge transfer and structural stability, while heteroatom co‐doping promoted active‐site exposure for both HER and OER. Similarly, Abdolahi et al. synthesized a Ni‐Co oxide/carbon hybrid from waste chicken feathers via solvothermal pyrolysis at 700°C, where the CFAC/Ni & Co oxides composite exhibited a cell potential of 1.71 V at 30 mA cm^−2^, maintaining excellent bifunctional activity [280]. The hierarchical porosity and S/N self‐doping of keratin‐derived carbon contributed to enhanced electron mobility and ion accessibility. Alongside it, rose‐petal‐derived Ni‐doped graphitic carbon (NGC) leverages a Murray‐network, in vivo Ni uptake to yield highly graphitic, Ni‐decorated carbons that act as bifunctional electrodes for OWS: in 1.0 M KOH the NGC‐1‖NGC‐0.1 cell shows an onset of 1.36 V and reaches 10 mA cm^−2^ at 1.64 V; as shown in Figure 10b, the device sustains electrolysis at 1.64 V for 24 h with only slight activity loss, underscoring transition‐metal‐carbon synergy (Ni active sites + high graphitization) for durable charge transport and gas‐evolution stability [271].

In another study, Lin et al. reported Co_2_P@CoP nanoparticles embedded in ginkgo‐leaf‐derived porous carbon, prepared by an ammonium‐nitrate‐assisted low‐temperature route [276]. The Co_2_P@CoP/PC catalyst delivered 1.63 V at 10 mA cm^−2^ with stability over 1000 min, where the Co_2_P@CoP heterointerface provided favorable hydrogen adsorption energy (ΔG_H* ≈ 0) and accelerated water dissociation kinetics. Expanding this approach, Zhang et al. utilized hazardous textile sludge to develop Cu_8_S_5_‐decorated N, S self‐doped porous carbon (Cu_8_S_5_/NSC‐900) via direct pyrolysis, achieving 10 mA cm^−2^ at 1.64 V, comparable to, or in some cases exceeding, Pt/C ‖ RuO_2_ benchmarks under identical testing conditions and current densities [272]. The improved performance originated from synergistic Cu_8_S_5_–carbon coupling and abundant mass‐transfer channels within the N, S‐doped carbon matrix. Furthermore, as shown in Figure 10c, Liu et al. fabricated a MoS_2_@NiOOH@C‐MC hybrid, where willow‐catkin‐derived mesoporous carbon served as a conductive scaffold supporting MoS_2_ and NiOOH nanosheets [282]. The resulting heterostructure delivered an impressive cell voltage of 1.62 V at 10 mA cm^−2^, outperforming Pt/C‖IrO_2_, while maintaining 98% current retention over 40 h. This superior bifunctional activity originated from the synergistic integration of MoS_2_'s hydrogen‐adsorption sites, NiOOH's oxygen‐evolution kinetics, and the highly conductive carbon backbone, ensuring efficient charge transfer and structural stability.

Overall, these studies reveal that transition‐metal‐carbon composites derived from renewable biomass offer a sustainable pathway toward efficient OWS electrocatalysts. The combination of metallic heterostructures (Co‐P, Ni‐Co‐O, Cu‐S, Mo‐S) and heteroatom‐doped carbon networks enables simultaneous HER/OER catalysis under low cell voltages (1.6–1.7 V). This enhanced performance arises from strong metal–carbon interfacial coupling, which modulates electronic structures and optimizes adsorption energies of reaction intermediates, while conductive carbon frameworks facilitate rapid charge transfer and porous architectures improve mass transport [80, 291, 292, 293, 294]. Comparative analysis indicates that heterostructure systems generally outperform single‐phase catalysts due to synergistic effects between multiple active sites. However, challenges remain in controlling heterointerface uniformity, phase stability under long‐term operation, and variability in biowaste‐derived structures affecting reproducibility [81, 291, 294, 295]. Future progress should emphasize controlled heterointerface engineering, defect modulation, and scalable synthesis to fully integrate these green materials within the circular bio‐economy hydrogen framework [273, 296], along with operando characterization and theoretical modeling to establish clear structure–activity relationships.

## 6 Stability, Mechanistic Insights, and Degradation Behavior

Although many biowaste‐derived electrocatalysts exhibit low *η* and favorable Tafel slopes, long‐term deployment in practical water electrolysis requires a deeper understanding of degradation pathways beyond initial catalytic activity. In particular, catalyst stability is governed not only by the intrinsic durability of the active phase, but also by the persistence of heteroatom dopants, the corrosion resistance of the carbon framework, and the structural integrity of metal–carbon interfaces under sustained HER/OER operation. These issues are especially critical for biowaste‐derived systems because their catalytic performance frequently arises from heteroatom‐rich defect structures and highly dispersed metal species, both of which may evolve dynamically under electrochemical polarization. Recent studies on OER stability emphasize that catalyst degradation commonly proceeds through surface reconstruction, dissolution, redeposition, lattice oxygen participation, and support degradation, all of which can substantially alter the initially identified active structure [251, 297, 298].

A key mechanistic concern for heteroatom‐doped biocarbons is whether dopants such as N, S, and P remain structurally stable during prolonged electrolysis. Increasing evidence suggests that under strongly anodic OER conditions, especially in alkaline or acidic oxidative environments, heteroatom functionalities can be progressively removed or transformed. Operando studies on N‐doped carbon catalysts have shown electrochemical denitrogenation during anodic polarization, indicating that N‐containing active motifs are not always permanent under working conditions. Likewise, a mechanistic study on heteroatom‐doped carbons under OER demonstrated that N‐, P‐ containing dopants can undergo near‐complete dissolution as high‐valence oxoanions, revealing that heteroatom leaching is not merely a surface cleaning process but can directly eliminate catalytically relevant doped sites. These findings are highly relevant to biowaste‐derived catalysts, where heteroatom‐induced charge redistribution is often invoked to explain activity. In such cases, apparent performance retention over short times may mask a gradual loss of chemically active dopant configurations. Therefore, future studies should verify dopant retention using post‐electrolysis XPS, elemental mapping, ICP analysis of the electrolyte, and, where possible, operando spectroscopies to distinguish genuine catalytic stability from transient reconstruction‐driven activity [298, 299].

Another important degradation route is carbon corrosion, particularly during OER at high anodic potentials. Carbon frameworks are often introduced into biowaste‐derived catalysts to improve conductivity, porosity, and metal dispersion; however, under sufficiently oxidative potentials, carbon can undergo electrochemical oxidation, leading to loss of conductivity, collapse of porosity, detachment of active particles, and destruction of heteroatom‐containing defect sites. This issue is most severe under acidic OER and at large current density, but it can also arise in alkaline environments over prolonged operation. Carbon corrosion has been directly linked to denitrogenation and structural degradation in electrochemical carbon materials, demonstrating that the carbon host cannot always be considered inert during OER. For biowaste‐derived carbons, which are frequently defect‐rich and partially graphitized rather than fully graphitic, this concern is even more pronounced because disordered carbon domains and edge‐rich structures are thermodynamically more vulnerable to oxidation. Thus, although defect engineering is beneficial for creating active sites, excessive disorder may compromise long‐term oxidative stability. This trade‐off implies that future design should balance catalytic defect density with sufficient graphitic stabilization, particularly for OER‐oriented or bifunctional electrodes [251, 299, 300].

For metal‐containing biowaste‐derived catalysts, degradation is further complicated by metal dissolution and interfacial instability. Transition metals such as Fe, Co, Ni, Mo, and Cu often serve as the true redox‐active centers, while the biowaste‐derived carbon functions as a conductive scaffold. Under HER conditions, dissolution is often less severe, although metal sulfides, phosphides, and carbides may still undergo phase conversion or surface amorphization. Under OER conditions, however, anodic potentials can induce partial dissolution of transition metals, dynamic reconstruction into oxyhydroxide phases, or migration of metal species away from their original coordination environment. Recent studies show that metal dissolution is not necessarily a secondary phenomenon; rather, it may occur concurrently with activation, surface restructuring, and active‐phase generation. This means that apparent improvement in OER activity over time may reflect the formation of a new active surface rather than genuine preservation of the original catalyst. For biowaste‐derived transition‐metal hybrids, these processes can weaken metal–support interactions, diminish active‐site density, and ultimately destabilize the catalyst architecture. Accordingly, long‐term analysis should include pre‐ and post‐electrolysis phase characterization, dissolved‐metal quantification in the electrolyte, and correlation of structural change with activity evolution [251, 297, 301, 302].

Taken together, current evidence suggests that stability in biowaste‐derived electrocatalysts should be interpreted through a reaction‐dependent degradation framework. For HER, the main concerns are generally phase retention, suppression of nanoparticle agglomeration, and stability of heteroatom‐modified adsorption sites. For OER, by contrast, the dominant challenges shift toward heteroatom leaching, carbon oxidation, metal dissolution, and irreversible reconstruction under oxidative bias. Consequently, future progress in biowaste‐derived catalysts should move beyond reporting only 10–20 h chronoamperometry or chronopotentiometry at modest current density. More informative stability evaluation should combine long‐duration electrolysis, accelerated degradation testing, electrolyte analysis, and operando or quasi‐operando structural characterization. Such an approach will allow researchers to determine whether activity is sustained by genuine structural robustness, by dynamic self‐reconstruction into a more active phase, or by gradual loss of the original catalytic motif. Establishing this mechanistic distinction is essential for translating biowaste‐derived catalysts from promising laboratory materials into practically durable electrodes for industrial water splitting [251, 262, 297, 303].

## 7 Conclusion and Future Perspectives

In conclusion, biowaste‐derived electrocatalyst for water splitting embodies a convergence of green chemistry, materials innovation, and circular‐economy principles. The current foundation demonstrates that sustainable hydrogen production can be achieved without reliance on critical metals or energy‐intensive precursors. At the same time, the best‐performing biowaste‐derived systems now rival many noble‐metal‐free benchmark catalysts in terms of *η*, Tafel slope, and durability, while offering substantially lower feedstock and processing costs. Importantly, the progress summarized in this review also confirms that catalyst performance is not determined solely by elemental composition, but by the coupled influence of precursor chemistry, pore architecture, heteroatom functionality, graphitic order, and metal–support interfacial structure. The next decade should focus on durability, standardization, and real‐device validation, supported by transparent sustainability metrics and predictive design tools. Through these coordinated efforts, biowaste valorization can evolve from laboratory curiosity to a mainstream technological pathway for clean and circular hydrogen generation. Following the principles of the circular bioeconomy, transforming biowaste into high‐efficiency electrocatalysts for water electrolysis offers both environmental and economic advantages by valorizing abundant organic residues into functional electrode materials. This review has comprehensively summarized recent progress in the development of biowaste‐derived electrodes for HER, OER, and OWS. Agricultural residues, food wastes, and marine biomasses have been successfully converted into heteroatom‐doped carbons and carbon‐metal hybrids through pyrolysis, hydrothermal treatment, chemical activation, plasma, or electrodeposition routes. These catalysts exhibit competitive activity owing to their hierarchical porosity, tunable electronic structure, and abundant active sites derived from intrinsic heteroatoms (N, S, P, B) and defect configurations. The collective evidence indicates that the electrocatalytic performance of biowaste‐derived materials is intimately governed by their precursor composition, nanostructure, and surface chemistry, which can be rationally controlled through synthetic design. At the same time, this review highlights that HER and OER impose fundamentally different catalytic demands: HER benefits more strongly from conductivity, graphitic order, and optimized hydrogen‐adsorption energetics, whereas OER requires greater emphasis on redox‐active surface chemistry, oxidative stability, and resistance to structural degradation. This distinction is especially important for the rational design of bifunctional electrodes, where activity alone is insufficient unless accompanied by interfacial robustness, scalable fabrication, and stable operation under practical current densities.

Despite these significant advancements, several research opportunities remain to be addressed to realize the full potential of biowaste‐derived electrodes in practical hydrogen production systems:

(1) **Diversifying and standardizing biomass feedstocks.** Current studies predominantly rely on common agricultural residues such as rice husk, coconut shell, or coffee waste. Future exploration should extend to under‐utilized or region‐specific biowastes (e.g, algal biomass, fruit‐processing residues, and marine shells) with unique heteroatom contents or hierarchical textures. Establishing standardized pretreatment and carbonization protocols will be essential to minimize batch variability and ensure reproducibility across laboratories.
(2) **Strengthening structure–activity correlation through advanced characterization.** The intrinsic complexity of biomass precursors leads to challenges in identifying the true active centers. To overcome this, operando techniques (X‐ray absorption, Raman, and EIS) combined with DFT simulations should be employed to elucidate the mechanistic role of heteroatom sites, vacancies, and metal–carbon interfaces. Particular attention should be devoted to monitoring the dynamic reconstruction of biowaste‐derived catalysts under realistic electrolysis conditions.
(3) **Engineering stability and scalability.** While biowaste‐based catalysts can deliver low *η* at 10–50 mA cm^−2^, maintaining performance at ≥100−300 mA cm^−2^ and for extended operation (>100 h) remains a critical target. Achieving this requires robust interface engineering, controlled heteroatom retention, and binder‐free, self‐supported architectures that suppress detachment and corrosion. Furthermore, developing low‐energy and continuous synthesis processes such as flash Joule heating, plasma activation, or one‐pot hydrothermal doping will be vital for industrial scalability.
(4) **Integrating circular metrics into catalyst evaluation.** Beyond electrochemical efficiency, the sustainability of biowaste‐derived electrodes should be quantified through life‐cycle assessment and techno‐economic analysis. Metrics such as carbon footprint per kilogram of hydrogen, feedstock utilization efficiency, and end‐of‐life recyclability must accompany performance benchmarking to validate their circular‐economy potential.
(5) **Extending multifunctionality and hybrid integration.** Given their tunable surface chemistry and redox versatility, biowaste‐derived materials could also serve as electrodes for paired electrolysis (e.g, biomass oxidation coupled with HER) or for other electrocatalytic conversions such as CO_2_ reduction, N_2_ fixation, and pollutant degradation. Such cross‐application studies will expand their technological relevance and accelerate the deployment of integrated, low‐carbon energy platforms.
(6) **Data‐driven discovery and predictive design.** Machine‐learning frameworks coupled with databases of biomass composition, synthesis conditions, and catalytic performance can reveal hidden correlations between precursor chemistry and activity descriptors. The adoption of AI‐assisted material design will expedite the identification of optimal doping ratios, pore architectures, and reaction environments for next‐generation biowaste electrodes.

## Author Contributions

**Vishal P. Bhandigare**: conceptualization, data curation, investigation, formal analysis, visualization, writing – original draft. **Jaydip K. Sawant**: data curation, investigation, formal analysis, visualization. **Sourabh B. Ghode**: data curation, investigation, formal analysis, visualization. **Jihyeon Kim**: data curation, investigation, visualization. **Chandrashekhar S. Patil**: data curation, investigation, formal analysis. **Charalampos Pitsalidis**: supervision, formal analysis. **Kyungsoon Park**: conceptualization, data curation, investigation, formal analysis, supervision, funding acquisition, writing – review and editing. **Jinho Bae**: conceptualization, formal analysis, supervision, funding acquisition, writing – review and editing.

## Funding

This study was supported by Jeju National University (2025).

## Conflicts of Interest

The authors declare no conflicts of interest.
